# A Metabolomic Analysis of Omega-3 Fatty Acid-Mediated Attenuation of Western Diet-Induced Nonalcoholic Steatohepatitis in *LDLR* ^*-/-*^ Mice

**DOI:** 10.1371/journal.pone.0083756

**Published:** 2013-12-17

**Authors:** Christopher M. Depner, Maret G. Traber, Gerd Bobe, Elizabeth Kensicki, Kurt M. Bohren, Ginger Milne, Donald B. Jump

**Affiliations:** 1 The Nutrition Program, School of Biological and Population Health Sciences, Oregon State University, Corvallis, Oregon, United States of America; 2 Department of Animal and Rangeland Sciences, Oregon State University, Corvallis, Oregon, United States of America; 3 The Linus Pauling Institute, Oregon State University, Corvallis, Oregon, United States of America; 4 Metabolon, Inc., Durham, North Carolina, United States of America; 5 United States Department of Agriculture, Agricultural Research Service, Children’s Nutrition Research Center, Baylor College of Medicine, Houston, Texas, United States of America; 6 Eicosanoid Core Laboratory, Division of Clinical Pharmacology, Vanderbilt University Medical Center, Nashville, Tennessee, United States of America; Wageningen University, Netherlands

## Abstract

**Background:**

Nonalcoholic steatohepatitis (NASH) is a progressive form of nonalcoholic fatty liver disease and a risk factor for cirrhosis, hepatocellular carcinoma and liver failure. Previously, we reported that dietary docosahexaenoic acid (DHA, 22:6,n-3) was more effective than eicosapentaenoic acid (EPA, 20:5,n-3) at reversing western diet (WD) induced NASH in LDLR^-/-^ mice.

**Methods:**

Using livers from our previous study, we carried out a global non-targeted metabolomic approach to quantify diet-induced changes in hepatic metabolism.

**Results:**

Livers from WD + olive oil (WD + O)-fed mice displayed histological and gene expression features consistent with NASH. The metabolomic analysis of 320 metabolites established that the WD and n-3 polyunsaturated fatty acid (PUFA) supplementation had broad effects on all major metabolic pathways. Livers from WD + O-fed mice were enriched in saturated (SFA) and monounsaturated fatty acids (MUFA), palmitoyl-sphingomyelin, cholesterol, n-6 PUFA, n-6 PUFA-containing phosphoglycerolipids, n-6 PUFA-derived oxidized lipids (12-HETE) and depleted of C_20-22_ n-3 PUFA-containing phosphoglycerolipids, C_20-22_ n-3 PUFA-derived oxidized lipids (18-HEPE, 17,18-DiHETE) and S-lactoylglutathione, a methylglyoxal detoxification product. WD + DHA was more effective than WD + EPA at attenuating WD + O-induced changes in NASH gene expression markers, n-6 PUFA and oxidized lipids, citrate and S-lactosyl glutathione. Diet-induced changes in hepatic MUFA and sphingolipid content were associated with changes in expression of enzymes involved in MUFA and sphingolipid synthesis. Changes in hepatic oxidized fatty acids and S-lactoylglutathione, however, correlated with hepatic n-3 and n-6 C_20-22_ PUFA content. Hepatic C_20-22_ n-3 PUFA content was inversely associated with hepatic α-tocopherol and ascorbate content and positively associated with urinary F2- and F3-isoprostanes, revealing diet effects on whole body oxidative stress.

**Conclusion:**

DHA regulation of hepatic SFA, MUFA, PUFA, sphingomyelin, PUFA-derived oxidized lipids and S-lactoylglutathione may explain the protective effects of DHA against WD-induced NASH in LDLR^-/-^ mice.

## Introduction

 Nonalcoholic steatohepatitis (NASH) is the progressive form of nonalcoholic fatty liver disease (NAFLD) and is defined as hepatic steatosis with inflammation and hepatic injury [1]. Once developed, NASH can progress to hepatic fibrosis, cirrhosis, hepatocellular carcinoma and end stage liver disease [2]. Approximately 30% to 40% of individuals with hepatic steatosis progress to NASH [2]; and the prevalence of NASH in the general population ranges from 3% to 5% [3]. Given its association with obesity, type 2 diabetes (T2D) and metabolic syndrome (MetS); and its increasing prevalence and clinical severity, NASH is quickly becoming a significant public health concern. NASH is estimated to be the leading cause of liver transplantation in the United States by 2020 [4].

 The development of NASH has been proposed to follow a two-hit mechanism [5]. The “1^st^ Hit” involves excess lipid accumulation in the liver, which sensitizes the liver to the “2^nd^ Hit”. The “2^nd^ Hit” involves inflammation, oxidative stress, liver damage and fibrosis. While the two-hit hypothesis is helpful in understanding processes that contribute to development and progression of NASH, our overall understanding of NASH is incomplete and thus has limited the development of therapies specifically targeted to NAFLD/NASH. The standard of care for patients diagnosed with NAFLD or NASH is to treat for liver disease and the associated co-morbidities including obesity, T2D, hyperlipidemia, and MetS [1,6,7]. As such, a more complete understanding of NASH is needed to address this imminent public health burden. 

 We recently reported that the capacity of dietary docosahexaenoic acid (DHA; 22:6,n-3) to suppress markers of hepatic damage (plasma alanine [ALT] and aspartate [AST] amino-transferases), hepatic inflammation (C-type lectin domain family 4-F [Clec4F], F4/80, toll-like receptor 4 [TLR4]), oxidative stress (NADPH oxidase subunits NOX2, p22phox, p40phox, p47phox, and p67phox), and fibrosis (pro-collagen 1A1 [proCol1A1], transforming growth-factor β1 [TGF β1]) was greater than dietary eicosapentaenoic acid (EPA, 20:5,n-3) using the LDLR^-/-^ mouse model of western diet (WD) induced NASH [8]. While dietary DHA provided significant benefit in preventing NASH progression, neither EPA nor DHA fully attenuated WD-induced hepatosteatosis. The outcome of this study established that a major target of DHA in the liver is the control of inflammation, oxidative stress, and fibrosis, the key features that distinguish NASH from benign steatosis. 

To advance our understanding of both the progression of NASH and the impact from EPA and DHA supplementation on NASH, we conducted a non-targeted global metabolomics analysis of livers from our previous study [8]. LDLR^-/-^ mice were fed the WD for 16 weeks with and without EPA and/or DHA supplementation. A major advantage of this study was that it provided an analysis of perturbed hepatic metabolism associated with advanced diet-induced NASH, the most clinically detrimental stage of NAFLD. Multiple studies have previously employed lipidomic and proteomic strategies to identify new NAFLD/NASH biomarkers [9-12]. Only a few studies have used metabolomics specifically on liver to assess changes associated with NAFLD or NASH [13-15]. As far as we are aware, this is the first study to use metabolomics to identify pathways involved in diet-induced NASH and also to evaluate the impact of dietary EPA and DHA on NASH-induced changes in hepatic metabolism. Our overall goal was to identify pathways contributing to NASH and assess the impact of EPA and DHA on the regulation of those pathways. Accordingly, we identified several metabolic targets of WD and C_20-22_ n-3 PUFA that potentially contribute to the development of NASH including sphingo- and phospho-glycerol lipid metabolism, membrane remodeling, oxidized lipid formation and methylglyoxal (MG) detoxification. MG is a metabolite involved advanced glycation end product formation and has been linked to NAFLD [16]. 

## Materials and Methods

### Animals and diets

All procedures for the use and care of animals for laboratory research were approved by the Institutional Animal Care and Use Committee at Oregon State University. Male LDLR^-/-^ mice [C57BL/6J background, Jackson Laboratories] at 2 months of age were fed one of the following five diets *ad libitum* for 16 weeks; each group consisted of 8 male mice. The control diet was Purina chow 5001 consisting of 13.5% energy as fat and 58.0% energy as carbohydrates. The western diet (WD) (D12709B, Research Diets] was used to induce NAFLD/NASH; it consists of 17% energy as protein, 43% energy as carbohydrate and 41% energy as fat; cholesterol was at 0.2% w/w. The WD was supplemented with olive oil (WD + O), EPA (WD + E), DHA (WD + D) or EPA plus DHA (WD + E + D). Supplementation of the WD with olive oil, EPA, DHA or EPA + DHA increased total fat energy to 44.7% and reduced protein and carbohydrate energy to 15.8 and 39.5%, respectively. Olive oil was added to the WD to ensure a uniform level of energy from fat, protein and carbohydrate in all WD diets. Preliminary studies established that the addition of olive oil to the WD had no effect on diet-induced fatty liver disease in LDLR^-/-^ mice. The C_20-22_ n-3 PUFA in the WD + E, WD + D and WD + E + D diets was at 2% total energy. A detailed analysis and description of the diets was reported previously [8]. At the end of the 16 week feeding period, all mice were fasted overnight (18:00 to 08:00 the next day) then euthanized (isoflurane anesthesia and exsanguination) at 08:00 for the collection of blood and liver [17]. 

### Urinary F2- and F3-isoprostanes (F2- & F3-IsoP)

After 15 weeks of feeding, 2 pairs of 3 mice from each diet group were placed in metabolic cages for 24 hour urine collections. Urine was stored at -80°C until analyzed for F2- and F3-isoprostanes. Results were normalized to urinary creatinine as previously described [17-19].

### RNA extraction and qRT-PCR

Total RNA was extracted from liver and specific transcripts were quantified by qRT-PCR [8,20]. Primers for each transcript are listed in Table **S1**. Cyclophilin was used as the internal control for all transcripts.

### Lipid extraction and analysis

Hepatic total lipid extracts were prepared as previously described [8,20]. Hepatic polar and neutral lipids were separated from the total lipid extracts by solid phase extraction using established methods [21,22]. Briefly, an Alltech aminopropyl disposable cartridge column was equilibrated with 2 column volumes of hexane. Total lipid extract in chloroform was loaded onto the column and washed with chloroform. Neutral lipids were eluted with chloroform-2-propanol (2:1). Non-esterified fatty acids (NEFA) were eluted with diethyl ether-2%acetic acid. Lastly, glycerophospholipids were eluted with methanol. Thin-layer chromatography was used to confirm separation of lipid classes (Figure **S1**)[8]. Fatty acid methyl esters were prepared from the polar, NEFA and neutral lipids and quantified by gas chromatography [8,20]. 

### Plasma Endotoxin

Plasma endotoxin was assayed using the Limulus Amebocyte lysate assay procedure according to the manufacturer’s instructions (Charles River). 

### Metabolomic analysis

The non-targeted global metabolomic analysis was carried out by Metabolon, Inc. (Durham, NC). Briefly, the sample preparation process was carried out using the automated MicroLab STAR® system from Hamilton Company. Recovery standards were added prior to the first step in the extraction process for quality control purposes. Sample preparation was conducted using a proprietary series of organic and aqueous extractions to remove the protein fraction while allowing maximum recovery of small molecules. The resulting extract was divided into two fractions; one for analysis by liquid chromatography (LC) and one for analysis by gas chromatography (GC). Samples were placed briefly on a TurboVap® (Zymark) to remove the organic solvent. Each sample was then frozen and dried under vacuum. Samples were then prepared for the appropriate instrument, either LC/MS or GC/MS.

 The LC/mass spectrometer (MS) portion of the platform was based on a Waters ACQUITY UPLC and a Thermo-Finnigan LTQ mass spectrometer, which consisted of an electrospray ionization (ESI) source and linear ion-trap (LIT) mass analyzer. The GC column was 5% phenyl and the temperature ramp was from 40° to 300° C in a 16 minute period. Samples were analyzed on a Thermo-Finnigan Trace DSQ fast-scanning single-quadrupole mass spectrometer using electron impact ionization.  Identification of known chemical entities was based on comparison to library entries of authentic standards. A detailed description of this platform has been published previously [13]. The metabolomic data is included as Supplementary Information (File **S1**).

 MultiExperiment Viewer (http://www.tm4.org) was used to analyze and represent the metabolomic data as a heat map. Detailed bioinformatic analysis was carried out using MetaboAnalyst (http://www.metaboanalyst.ca). The original metabolomics data was generated from analysis of mass equivalent-samples. However, to account for differential cellular protein content arising from the massive accumulation of lipid in livers, the original data was normalized to protein abundance/sample. In addition, gene expression data (delta C_t_ values) from our previous study [8] was added to the metabolomics data file. These transcripts serve as markers for NASH and lipid metabolism and include: monocyte chemoattractant protein-1 (MCP1), cluster of differentiation 68 (CD68), NADPH-oxidase-2 (NOX2), toll-like receptor-4 (TLR4), procollagen1A1 (proCOL1A1), and stearoyl CoA desaturase-1 (SCD1). This analysis allowed us to visualize how metabolites and NASH gene expression markers (transcripts) changed with treatment. The metabolomic analysis (MetaboAnalyst (http://www.metaboanalyst.ca) included several statistical analyses, i.e., principal component analysis (Figure **S2**), volcano plots, partial least squares-discriminant analysis, significance analysis of microarray, empirical Bayesian analysis of microarray, hierarchical clustering, random forest. 

### Statistical analysis

Changes in metabolites, proteins (immunoblot), endotoxin, and gene transcripts were analyzed by one-way ANOVA to detect significant differences between groups. Data were analyzed for homogenous variances by the Levine test. If unequal variances were detected, data were log-transformed. ANOVA analysis was performed on both transformed and untransformed data. A *p*-value < 0.05 was considered significantly different. Values are reported as mean ± SD.

## Results

### Overview of diet effects on hepatic metabolites

LDLR^-/-^ mice fed the WD + O for 16 weeks developed a robust NASH phenotype characterized by hepatosteatosis, hepatic damage (plasma ALT & AST), inflammation (MCP1), oxidative stress (Hmox-1 and NOX2) and fibrosis (ProCol1A1). While including C_20-22_ n-3 PUFA at 2% total energy in the WD did not fully prevent hepatosteatosis, NASH markers of hepatic damage, inflammation, oxidative stress, and fibrosis, were significantly attenuated. The WD + D diet was more effective than the WD + E diet at reversing WD + O induced NASH markers [8].

 To gain additional insight into how the WD + O and dietary C_20-22_ n-3 PUFA affected hepatic metabolism, we carried out a non-targeted global metabolomic analysis using livers from our previous study [8]. The analysis identified 524 total metabolites; 320 known and 204 unknown metabolites. When examined by principal component analysis, the 320 known metabolites in mice fed the chow diet had considerable variation in composition, while metabolites in the WD + D group differed from the other WD diets. Also, the WD + O differed from WD + E and WD + E + D in the second principal component (Figure **S2**). 

**Figure 1:**
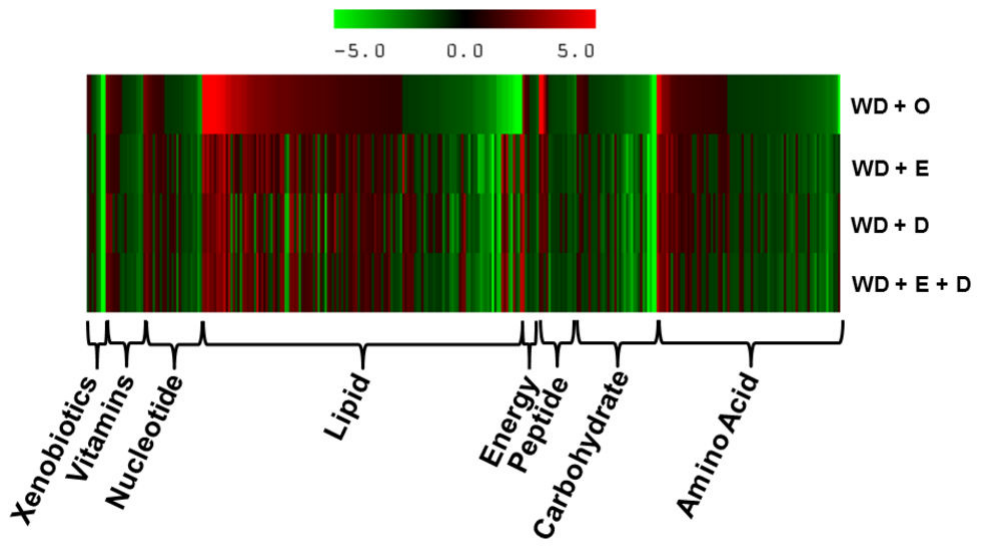
Heat map of diet effects on liver. The heat map represents the fold-change for each metabolite relative to control chow-fed versus WD-fed mice. The WD was supplemented with olive (O), EPA (E), DHA (D) or EPA and DHA (E + D). Results are sorted by fold-change within each pathway.

**Figure 2:**
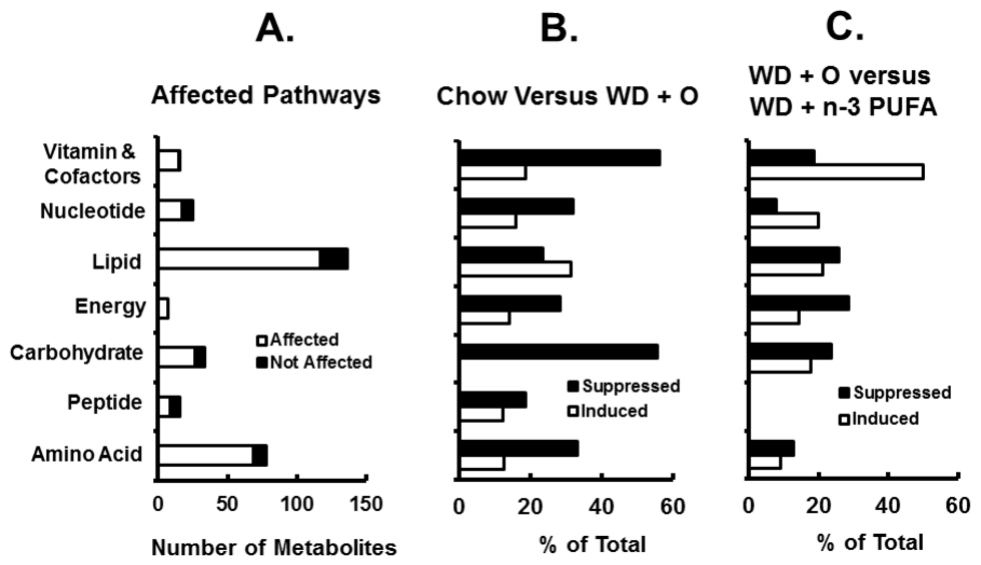
Diet effects on metabolic pathways. Panel A: Number of metabolites significantly changed in each pathway by all diets. Panel B: Percent of metabolites in each pathway that were induced or suppressed in WD + O fed mice relative to chow fed mice. Panel C: Percent of metabolites in each pathway that were induced or suppressed in all WD + C_20-22_ n-3 PUFA fed mice relative to WD + O fed mice.

A heat map of the 320 known metabolites is presented as fold-change relative to chow-fed (control) mice (Figure **1**). The known metabolites were associated with 8 major pathways including amino acid, carbohydrate, energy, lipid, nucleotide, peptide, vitamins and cofactors, and xenobiotics; xenobiotics (8 metabolites) were excluded from further analysis. The analysis revealed that metabolites associated with lipid and amino acid pathways were most affected by diet (Figure **2A**). Over 50% of the metabolites in each pathway were significantly affected by the dietary treatments. When compared to chow-fed mice, the WD + O diet affected all pathways; suppression of metabolite abundance was more common in the amino acid, carbohydrate, energy, nucleotide, and vitamin & cofactor pathways (Figure **2B**). Dietary supplementation with EPA and or DHA attenuated many of the WD + O-induced effects (Figure **2C**). Clearly the WD, without and with EPA or DHA supplementation has broad effects on liver metabolism affecting all major pathways. 

**Figure 3:**
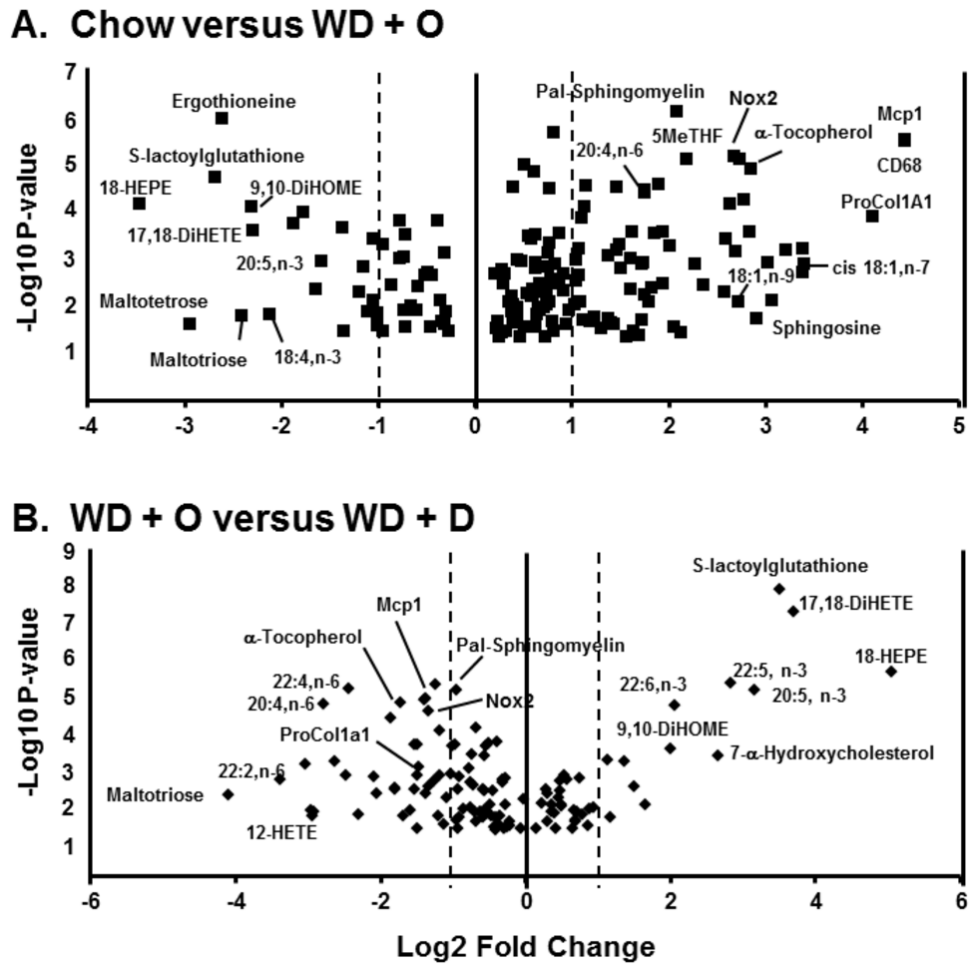
Volcano plots of diet effects on hepatic metabolites. Volcano plots were prepared using MetaboAnalyst (http://www.metaboanalyst.ca). The following groups (8 mice/group) were examined, Panel A: Chow versus WD + O; Panel B: WD + O versus WD + D. Results were plotted as log2 fold-change versus -log10 p-value. Several metabolites are labeled in each plot.

Since feeding mice the WD + D was better than WD + E at reversing WD + O effects on the liver [8], we prepared a volcano plot comparing chow-fed versus WD + O-fed mice and WD + O-fed versus WD + D-fed mice (Figure **3**). Volcano plots allow for the visualization of the distribution of p-values versus fold-change for all known metabolites and selected RNA transcripts [Tables **S2** - **S3** for the full volcano plot data]. Feeding mice the WD + O for 16 weeks led to an accumulation of palmitoyl-sphingomyelin, MUFA (18:1,n-9 and 18:1,n-7), n-6 PUFA (20:4, n-6) as well as vitamins or their metabolites, including α-tocopherol (vitamin E) and 5-methyl tetrahydrofolate (5MeTHF) (Figure **3A**). Feeding mice the WD + O diet also lowered hepatic n-3 PUFA (EPA, DHA) and oxidized lipids derived from n-3 PUFA (18-hydroxyeicosapentaenoic acid [18-HEPE] and 17,18-dihydroxyeicosatetraenoic acid [17,18-DiHETE]). Diet-induced changes in hepatic MUFA, n-3 and n-6 PUFA content paralleled changes reported previously using gas chromatographic analysis of total hepatic fatty acid content [8]. Ingestion of the WD + O diet was also associated with the loss of S-lactoylglutathione, a detoxification product of methylglyoxal (MG); MG is involved in forming advanced glycation end products (AGEP) [23] and promotes NASH [16]. These changes in hepatic metabolites were associated with hepatic damage (ATL and AST) and the induction of multiple gene expression markers of NASH, including MUFA synthesis (SCD1), inflammation (MCP1, CD68, TLR4), oxidative stress (HMOX-1 & NOX2) and fibrosis (proCOL1A1). 

The comparison of WD + O versus WD + D (Figure **3B**) shows that many of the metabolites that changed in response to the WD + O diet (Figure **3A**) were reversed (partially or totally) by the WD + D. For example, hepatic C_20-22_ n-3 PUFA and their oxidized lipid metabolites increased, as did S-lactoylglutathione. Hepatic MUFA, n-6 PUFA, n-6 PUFA-derived oxidized lipids, palmitoyl-sphingomyelin and α-tocopherol, in contrast, were decreased in livers of WD + D-fed mice. Changes in hepatic content of these metabolites are associated with a corresponding decline in the NASH gene expression markers, i.e., MCP1, CD68, ProCOL1A1, NOX2, SCD1 and TLR4. 

### Plasma endotoxin and hepatic palmitoyl-sphingomyelin

**Figure 4:**
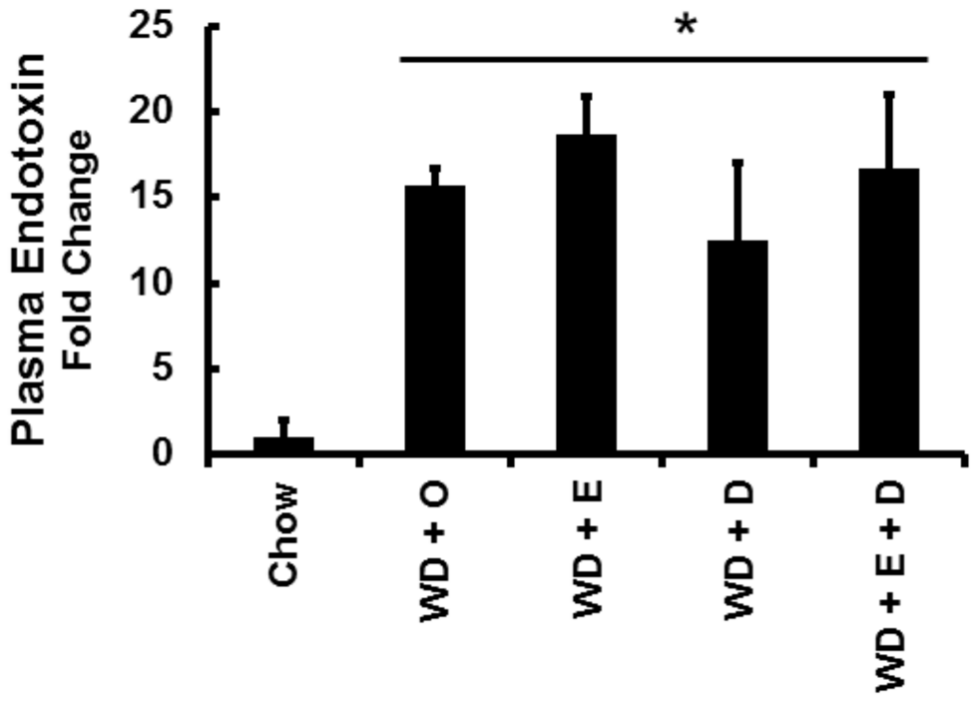
Diet effects on plasma endotoxin. Plasma endotoxin was quantified as described in Materials and Methods. Results are presented as Plasma Endotoxin, Fold Change. Mean ± SD relative to chow fed mice; *, *P* ≤ 0.05 versus chow.

Monocyte chemo-attractant protein-1 (MCP1) represents an early and robust marker of inflammation; MCP1 mRNA was induced >30-fold by feeding LDLR^-/-^ mice the WD + O [8]. There are several sources of inflammatory signals that impact the liver including oxidized LDL (ox-LDL) [24], endotoxin [25,26] and products from hepatocellular death resulting from hepatic injury [27,28]. In this report, we focused on endotoxin, which originates from the gut either by increased gut permeability or co-transport with chylomicron [26,29,30]. Plasma endotoxin of mice fed the WD + O is ~15-fold (p<0.05) higher than mice fed chow (Figure **4**). Plasma endotoxin is well-induced in all groups fed the WD, regardless of the absence or presence of dietary C_20-22_ n-3 PUFA. 

**Figure 5:**
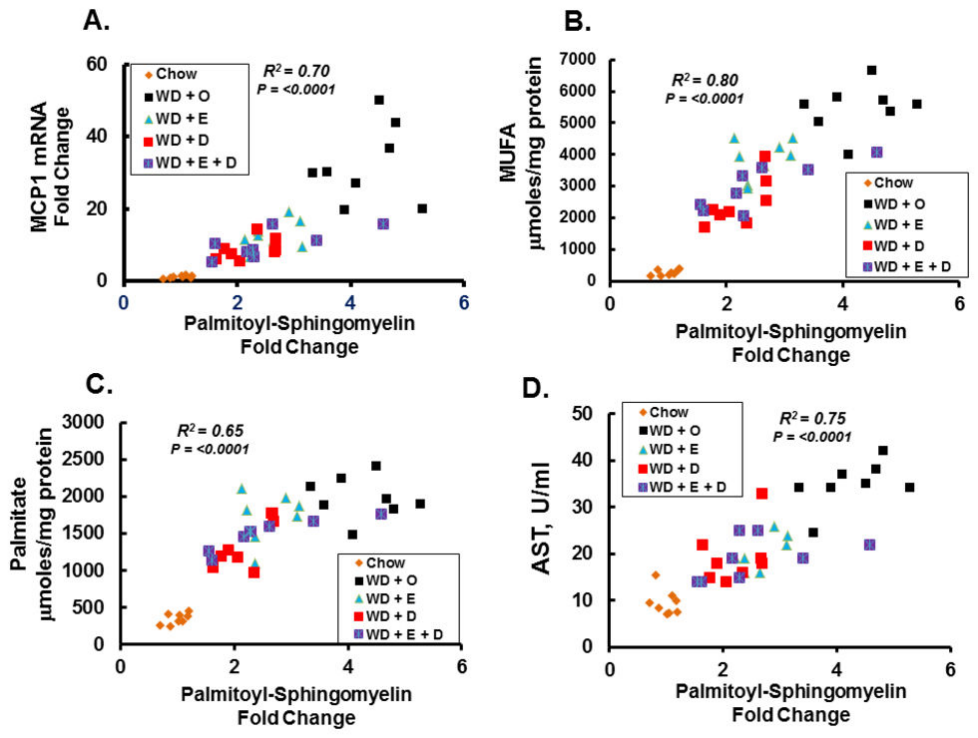
Diet effects on hepatic markers of inflammation, SFA, MUFA and damage. Linear regression analysis of hepatic palmitoyl-sphingomyelin (Fold Change relative to chow) versus hepatic MCP1 mRNA expression (fold change relative to chow) (Panel A); hepatic total MUFA content (µmoles total MUFA/mg protein) (Panel B); hepatic palmitate (16:0) (µmoles /mg protein) (Panel C); and plasma AST (units (U)/ml of plasma) (Panel D). Palmitoyl-sphingomyelin was quantified in the metabolomic analysis while hepatic MCP1, MUFA, palmitate, and plasma AST were quantified and reported previously [8]. Each data point in Panels A-D represents the relative abundance of palmitoyl-sphingomyelin and hepatic MCP1 mRNA, MUFA, 16:0 or plasma AST for each animal. The groups are colored-coded to facilitate visualization of the distribution in each group.

CD14 is the cellular receptor for endotoxin and it is linked to toll-like receptor-4 (TLR4) function [31]. Activation of TLR4 regulates a pathway that results in the accumulation of NFκB (p50 & p65 subunits) in the nucleus. MCP1 is one of many gene targets of NFκB [8]. TLR4 functions within lipid rafts, membrane microdomains enriched in sphingomyelin, cholesterol and phospholipids with saturated acyl chains [31]. Changes in hepatic palmitoyl-sphingomyelin correlated well with hepatic MCP1 expression, hepatic total MUFA, palmitate (16:0) and hepatic damage (plasma AST) (Figure **5 A-D**). DHA- and EPA-containing diets appear equally effective at reversing WD + O-induced changes in hepatic palmitoyl-sphingomyelin, MUFA, SFA and plasma AST.

**Figure 6:**
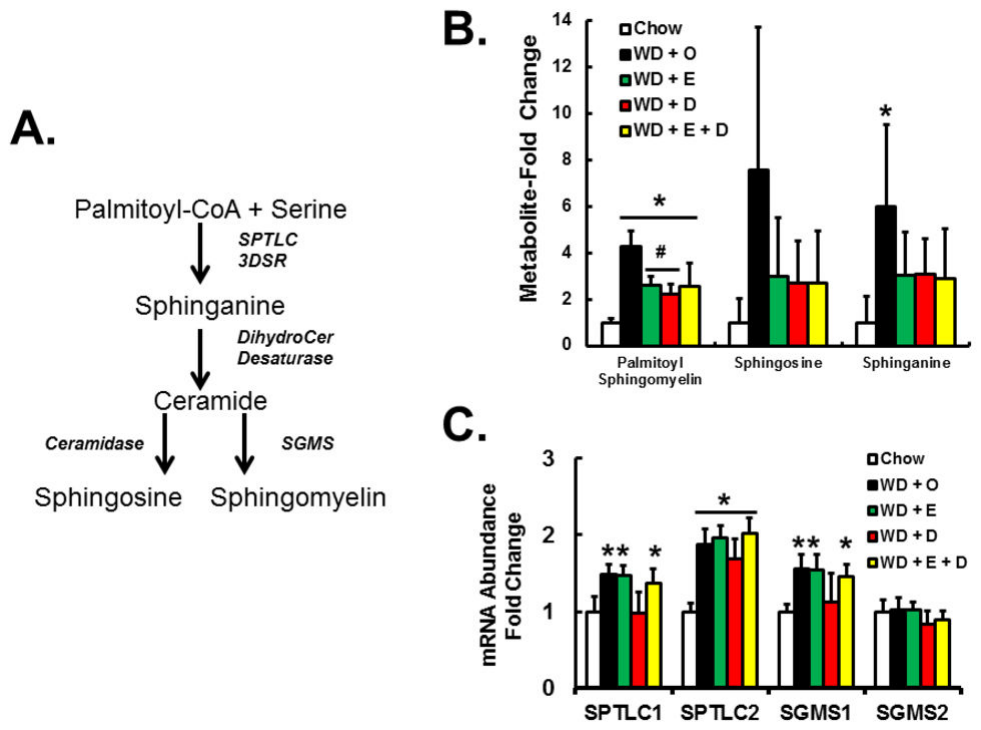
Diet effects on sphingolipid metabolites and enzymes involved in sphingolipid synthesis. Panel A: Pathway for *de* *novo* sphingomyelin synthesis. Panel B: Hepatic palmitoyl-sphingomyelin and metabolites involved in sphingomyelin synthesis (sphinganine) and ceramide degradation (sphingosine). Results are represented as Metabolites-Fold Change relative to chow; mean ± SD, n=8 per group. Panel C: RNA expression of key enzymes involved in sphingomyelin synthesis; mean ± SD. [serine-palmitoyl transferase long chain base subunit-1 & 2 (SPTLC1 & 2) and phosphatidylcholine:ceramide choline phosphotransferase 1 & 2 (SGMS1 & 2)]; *, *p* ≤ 0.05 versus chow; #, *p* ≤ 0.05 versus WD + O.

 Hepatic content of palmitoyl-sphingomyelin and metabolites involved in sphingomyelin synthesis (sphinganine) and ceramide degradation (sphingosine) were elevated in mice fed the WD + O diet (Figure **6 A-C**) suggesting effects on both synthesis of sphingomyelin and ceramide degradation. We examined the expression of enzymes involved in sphingomyelin synthesis, including serine-palmitoyl transferase long chain base subunit-1 & 2 (SPTLC1 & 2) and phosphatidylcholine:ceramide choline phosphotransferase 1 & 2 (SGMS1 & 2) (Figure **6C**). Of these, hepatic mRNA levels of SPTLC1, SPTLC2 and SGMS1 were induced by WD + O. WD +D, but not WD + E, blocked the WD + O induction of SPTLC1 and SGMS1, but not SPTLC2. While the involvement of sphingolipid synthesis in the progression NAFLD has been documented [32], this analysis suggests DHA modifies hepatic sphingolipid levels, at least in part, by controlling sphingolipid synthesis. We suggest these changes in hepatic sphingomyelin alter membrane lipid composition and TLR4 signaling. 

### One-carbon metabolism

**Figure 7:**
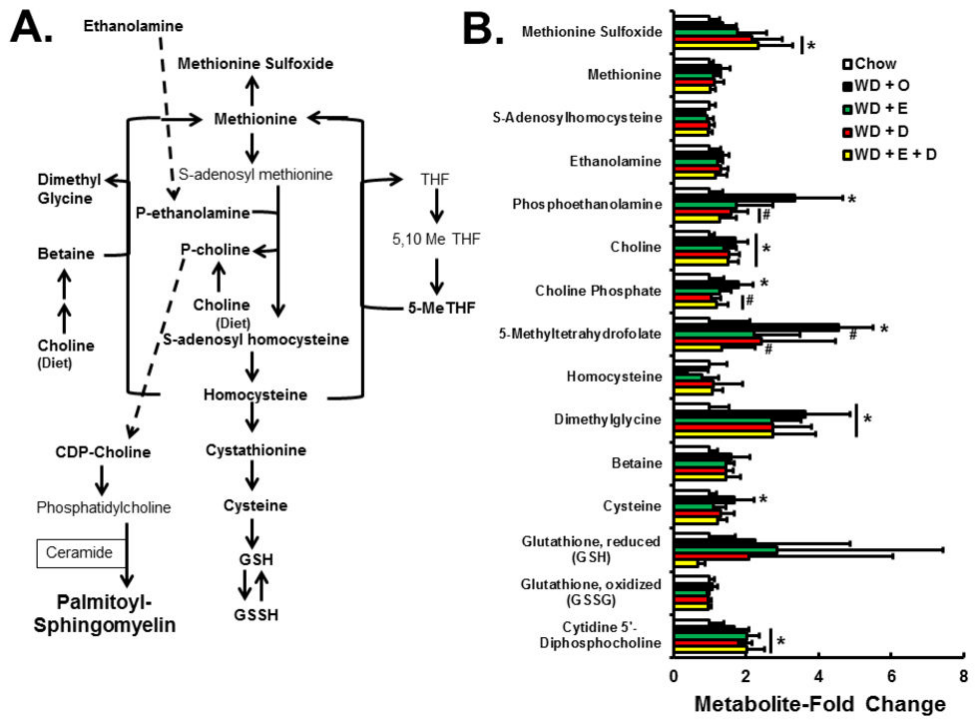
Diet effects on hepatic one-carbon, choline and glutathione metabolism. Panel A: Pathways for one-carbon, choline, glutathione and sphingomyelin metabolism. Panel B: Metabolites quantified by the metabolomic analysis were expressed as Metabolite-Fold Change and represented as mean ± SD, n=8 per group; *, *p* ≤ 0.05 versus chow; #, *p* ≤ 0.05 versus WD + O.

A key component of sphingomyelin is choline, an essential nutrient (Figure **7A & B**). Quantitation of metabolites involved in choline metabolism showed that the WD + O diet increased formation of phosphoethanolamine, choline, choline phosphate, 5MeTHF, dimethylglycine, cysteine, and cytidine 5’-diphosphocholine (CDP-choline). EPA and/or DHA containing diets attenuated the WD + O-induced changes in phosphoethanolamine, choline phosphate, and 5MeTHF. One explanation is that increased choline used for sphingomyelin synthesis in WD + O fed mice was prevented by decreased sphingomyelin synthesis in WD + D-fed mice. 

 Also quantified in this analysis are the oxidative stress markers associated with this pathway; including reduced (GSH) and oxidized (GSSH) glutathione and methionine sulfoxide. Of these metabolites, only methionine sulfoxide was significantly elevated in livers of mice fed WD + E and WD + D. These results suggest that EPA and DHA have selective effects on hepatic oxidative stress, affecting hepatic levels of methionine sulfoxide, but not GSH or GSSH. 

### Saturated (SFA) and monounsaturated fatty acids (MUFA)

**Figure 8:**
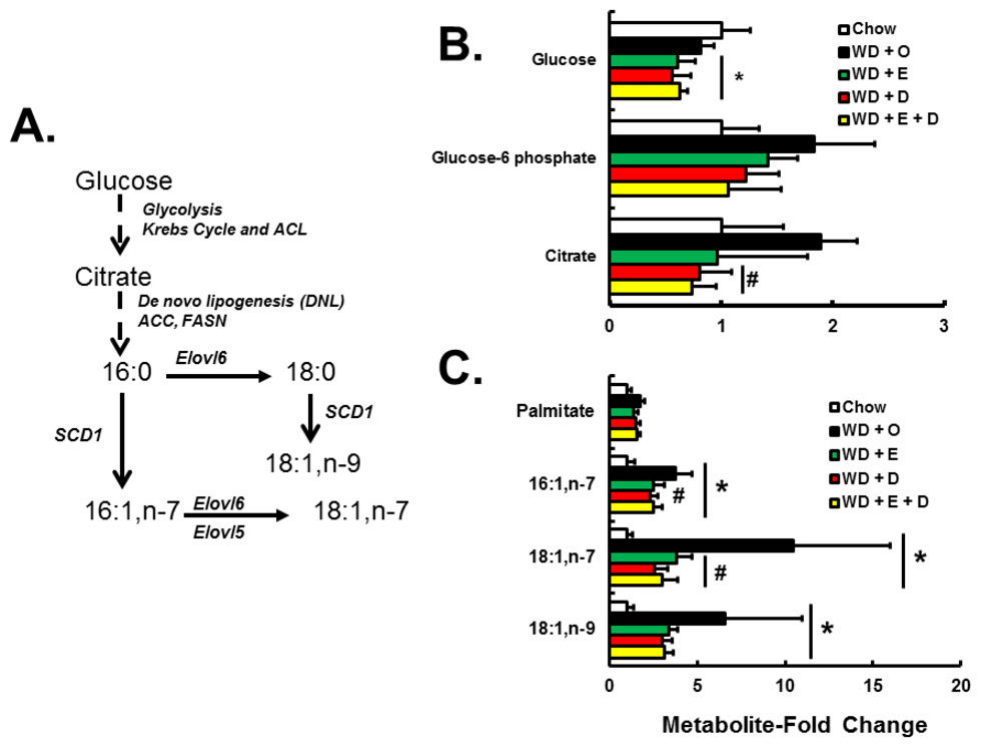
Diet effects on glucose metabolism and *de* *novo* MUFA synthesis. Panel A: Glucose conversion to saturated and monounsaturated fatty acids. Panel B: Metabolites involved in glucose metabolism were quantified by the metabolomic analysis and expressed as Metabolite-Fold Change; mean ± SD, n=8 per group. Panel C: Metabolites involved in *de* *novo* MUFA synthesis were quantified by the metabolomic analysis and expressed as Metabolite-Fold Change; mean ± SD, n=8 per group; *, *p* ≤ 0.05 versus chow; #, *p*≤ 0.05 versus WD + O.

Hepatic SFA and MUFA content correlates with increased palmitoyl-sphingomyelin (Figure **5**). The WD contains high levels of SFA and MUFA and feeding LDLR^-/-^ mice the WD + O diet leads to ~6-fold accumulation of 16:0 and >10-fold accumulation of 18:1,n-9 and 18:1,n-7 in the liver [8]. The WD + O diet also induced enzymes involved in MUFA synthesis, including SCD1 as well as two fatty acid elongases, ELOVL5 and ELOVL6 [8], suggesting that the massive accumulation of MUFA was due to both diet and *de novo* SFA and MUFA synthesis. The metabolomic analysis indicated that hepatic citrate was elevated in livers of WD + O fed mice versus livers of chow fed mice (Figure **8**). Increased levels of citrate inhibit phosphofructokinase-1, and therefore glucose-6 phosphate levels trend higher in WD + O fed mice. Increased hepatic citrate also supports increased *de novo* lipogenesis (DNL) which leads to the accumulation of 16:0, 18:1,n-9, and 18:1,n-7. DHA-containing diets significantly lowered hepatic citrate when compare to WD + O fed mice. 

**Figure 9:**
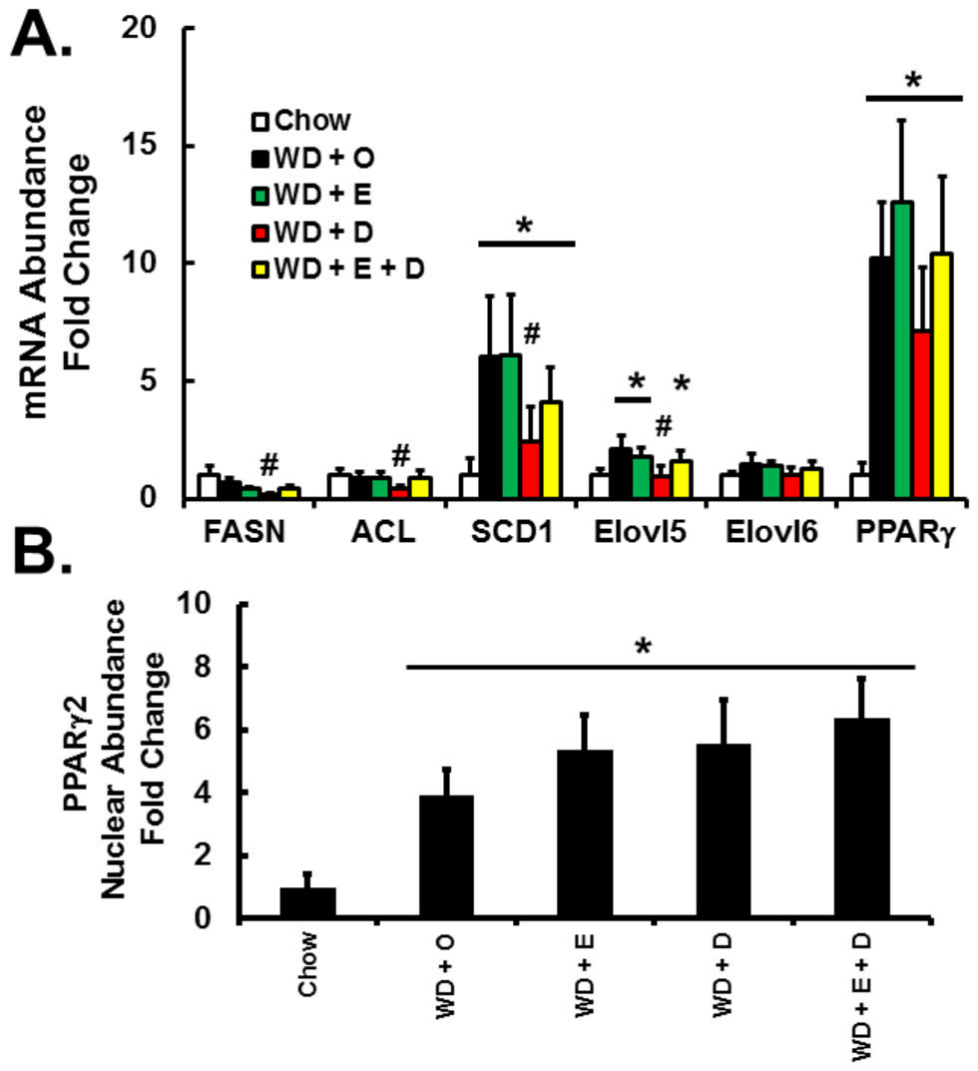
Diet effects on the expression of PPARγ2 and enzymes involved in MUFA synthesis. Panel A: Expression of enzymes involved in MUFA synthesis and the nuclear receptor peroxisome proliferator activated receptor γ2 (PPARγ2). Results are represented as mRNA Abundance-Fold Change, relative to chow-fed mice; mean ± SD, n=8/group. [Fatty acid synthase (FASN); ATP citrate lyase (ACL); stearoyl CoA desaturase 1 (SCD1), fatty acid elongase (Elovl)] Panel B: Hepatic nuclear abundance of PPARγ2 expressed as Fold Change relative to chow-fed mice; mean ± SD; n=8/group; *, *P* ≤ 0.05 versus chow; #, *P* ≤ 0.05 versus WD + O.

 SCD1 is a key enzyme involved in hepatic MUFA synthesis and its expression is regulated by multiple transcription factors, including sterol regulatory element binding protein (SREBP1), carbohydrate response element binding protein (ChREBP), liver X receptor (LXR), and peroxisome proliferator activated receptor γ2 (PPARγ2) [33]. Furthermore, regulation of SCD1 has been linked to SPTLC activity and has been implicated in sphingomyelin synthesis [34]. We previously reported the effects of a high-fat high-cholesterol diet and addition of C_20-22_ n-3 PUFA on SREBP1 and ChREBP nuclear abundance [17]. Here we show that SCD1 mRNA parallels the induction of hepatic PPARγ2 mRNA and nuclear abundance in WD + O fed mice (Figures **9A & B**). None of the diets containing EPA or DHA blocked the WD-mediated induction of nuclear PPARγ2. Nevertheless, DHA, but not EPA, suppressed SCD1 expression. DHA is a robust inhibitor of SREBP1 nuclear abundance [35]. Thus, DHA likely attenuates MUFA synthesis by decreasing fatty acid synthase (FASN), ATP-citrate lyase (ACL) and SCD1 expression. Taken together, these results suggest that DHA-containing diets regulate hepatic 16:0, 18:1,n-7, and 18:1,n-9 content by controlling multiple genes involved in DNL and MUFA synthesis as well as the availability of substrates required for DNL. DHA suppression of DNL and MUFA synthesis, however, is not achieved by suppression of hepatic nuclear abundance of PPARγ2 (Figures **8** & **9**). 

### Phospholipids and membrane remodeling

**Figure 10:**
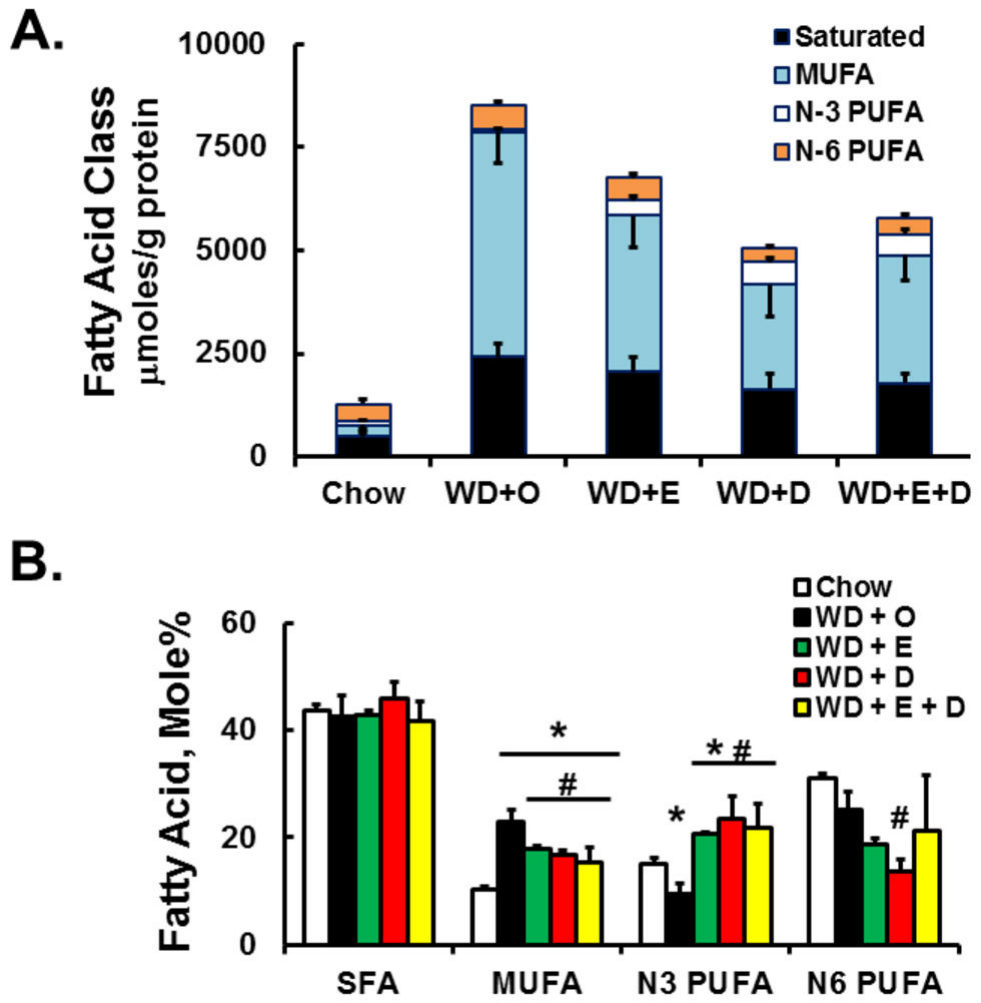
Diet effects on hepatic fat content. Panel A: Abundance of total hepatic SFA, MUFA, n-6 PUFA, and n-3 PUFA fatty acids analyzed from total lipid extracts using gas chromatography [8]. The sum of the fatty acids in each group (SFA, MUFA, n-3 and n-6 PUFA) is presented to illustrate the cumulative effects of diet on hepatic fat. Results are presented as total µmoles of fatty acid/g protein; mean + SD in each fatty acid class; n=8/group. Panel B: Distribution of SFA, MUFA, n-6 PUFA, and n-3 PUFA in hepatic phospholipids. Hepatic phospholipids were fractionated by solid phase separation, saponified and methylated for GC analysis (see Fig, S1 for the quality of the separation of phospholipids from total lipids). Results are expressed as Fatty Acid Mole% in each fatty acid class, i.e., SFA, MUFA, N3 and N6-PUFA; mean + SD, n=8/group.

**Figure 11:**
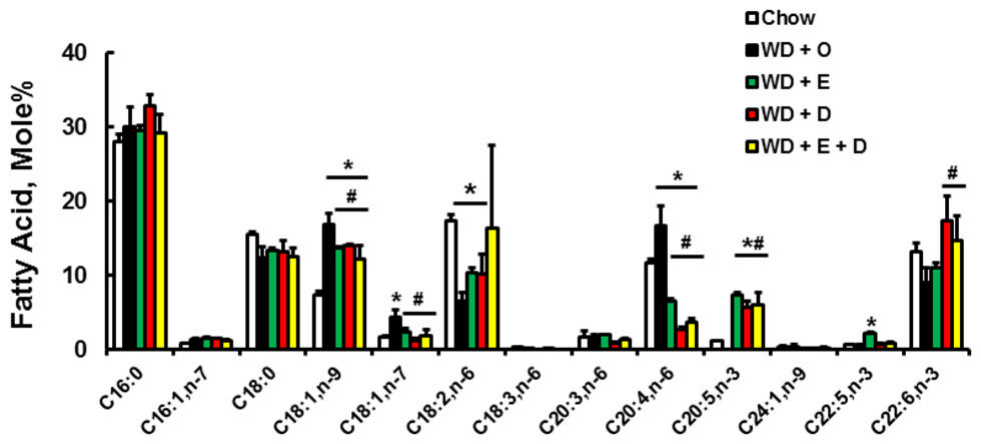
Diet effects on hepatic phospholipid fatty acids. Individual fatty acids in the phospholipid fraction were quantified as described above and represented as Fatty Acid-Mole%, mean + SD, n=8/group; *, *P* ≤ 0.05 versus chow; #, *P* ≤ 0.05 versus WD + O.

Feeding LDLR^-/-^ mice the WD + O leads to a massive increase in total hepatic fat, composed predominantly of SFA and MUFA (Figure **10A**). To examine the impact of this massive change in hepatic fat on membrane composition, we isolated hepatic phospholipids and examined the acyl chain composition (Figure **10B**; Figure **S1**). When expressed as mole%, the sum of all SFAs in the phospholipid fraction was not affected by diet. In contrast, WD + O feeding elevated the sum of all MUFA in the phospholipid fraction by >100% (*P* < 0.05); MUFA was decreased significantly by the WD + E and WD + D diets, probably because of effects on DNL and MUFA synthesis described above. The WD + O diet significantly decreased n-3 PUFA in phospholipids, while the WD + E and WD + D diets significantly increased n-3 PUFA in phospholipids. The EPA and DHA-containing diets significantly decrease n-6 PUFA in phospholipids. Changes in phospholipid fatty acid composition are illustrated in Figure **11**. Fatty acids most affected by diet were the MUFA (18:1, n-7 and n-9) and PUFA (20:4,n-6, 20:5,n-3 and 22:5,n-3). DHA in the phospholipid fraction was only elevated by DHA containing diets. 

**Figure 12:**
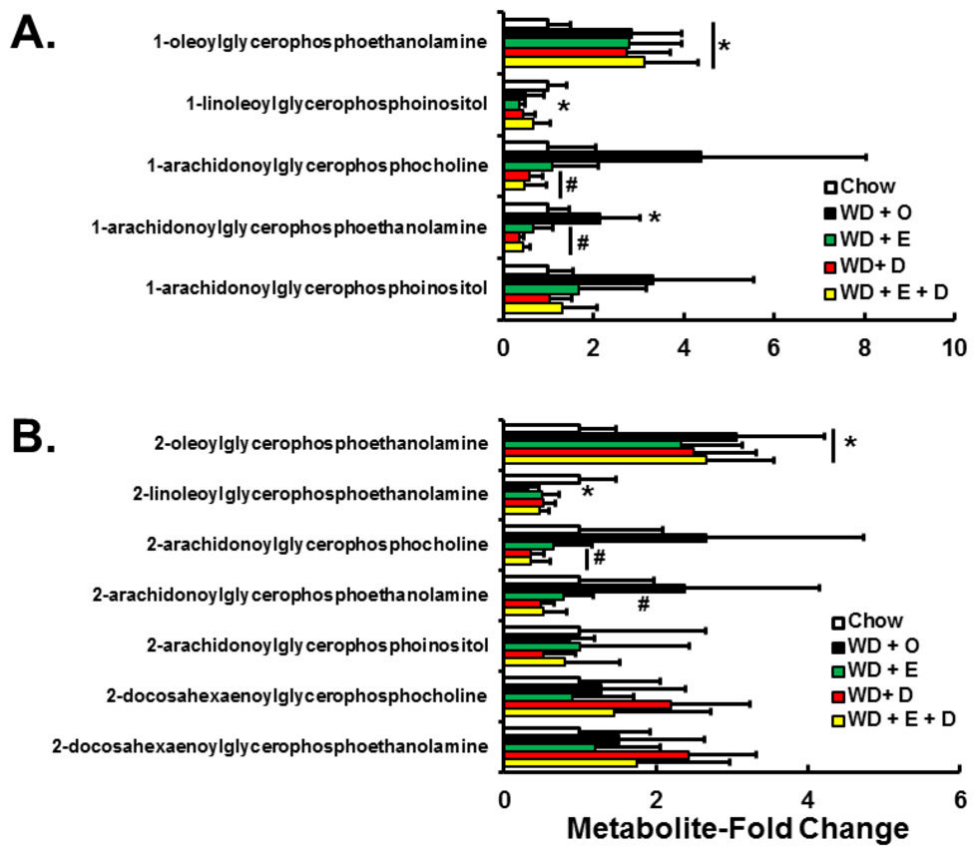
Diet effects on hepatic lysophospholipids. Panels A and B: Lysophospholipids were fractionated and quantified by the metabolomic analysis (LC/MS) as described in the Methods. Panel A: Lysophospholipids with acyl chains in the sn-1 position; Panel B: Lysophospholipids with acyl chains in the sn-2 position. The lysophospholipids are represented as Metabolite-Fold Change relative to chow; mean ± SD; n=8/group; *, *P* ≤ 0.05 versus chow; #, *P* ≤ 0.05 versus WD + O.

 The metabolomic analysis identified 42 lysophospholipids. Lysophospholipids are generated during *de novo* phospholipid synthesis or during membrane remodeling. Of the 26 lysophospholipids with acyl chains in the sn-1 position, 4 were significantly affected by diet (Figure **12A**). When compared to chow, 1-oleoylphosphoethanolamine was increased ~3-fold (p<0.05) in livers of mice fed the WD + O; the presence and absence of C_20-22_ n-3 PUFA in WD had no effect on this change. One-linoleoylphosphoinositol was decreased by ~50% (p<0.05) by WD + E, only. Levels of 1-arachidonylphosphocholine and –ethanolamine, but not –inositol were increased by the WD + O diet, while diets containing EPA or DHA decreased these lysophospholipids by >50% (p<0.05). 

 Of the 16 identified lysophospholipids with acyl chains in the sn-2 position, 4 were significantly affected by diet (Figure **12B**). Hepatic 2-oleoylglyceroethanolamine was increased ~3-fold (p<0.05) by WD + O regardless of the presence and absence of EPA and DHA, while hepatic levels of 2-linolenoylphosphoethanolamine were suppressed >50% (p<0.05) by WD + O. When compared to WD + O fed mice, hepatic levels of 2-arachidonylphosphoglycerol-choline and –ethanolamine, but not -inositol, were suppressed by >60% (p<0.05). Hepatic levels of lysophospholipids containing DHA were not affected by diet. 

**Figure 13:**
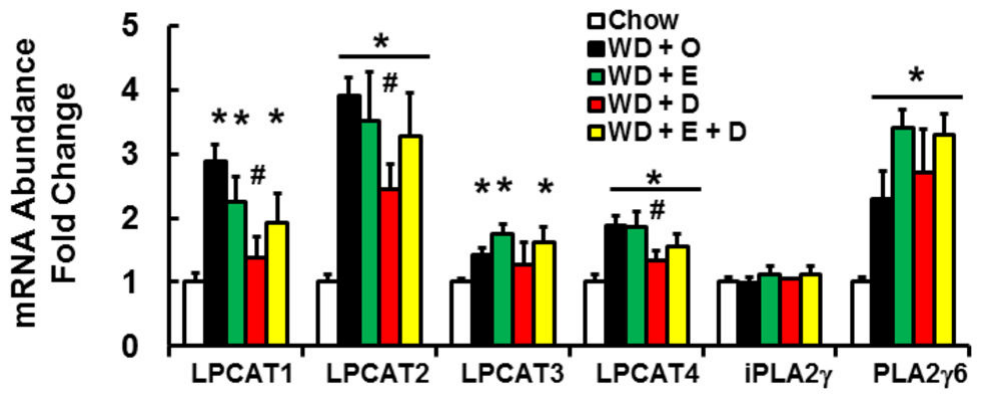
Diet effects on the expression of enzymes involved in membrane remodeling. Expression of enzymes involved in the incorporation of fatty acyl chains into phospholipids (lysophosphatidylcholine acyl transferase subtypes, LPCAT1-4) and excision of fatty acids from the sn-2 position of phospholipids (phospholipase A2 subtypes, iPLA2γ and PLA2γ6) were quantified. Results are represented as mRNA Abundance-Fold Change, mean ± SD; n=8/group; *, *p* ≤ 0.05 versus chow; #, *p* ≤ 0.05 versus WD + O.

 This analysis revealed significant changes in lysophospholipids containing oleic, linolenic and arachidonic, but not DHA. This outcome suggests significant changes in either *de novo* phospholipid synthesis or remodeling of membrane phospholipids. We examined the expression of enzymes involved in membrane remodeling; including four lysophosphatidylcholine acyl transferase subtypes (LPCAT1-4) and two phospholipase subtypes (iPLA2γ and PLA2γ6) (Figure **13**). WD + O induced transcripts encoding all LPCAT subtypes (> 30%), while WD + D suppressed LPCAT1, 2, and 4 expression. The phospholipase, iPLA2γ, was unaffected by diet, while PLA2γ6 was induced (~2-fold, p<0.05) by the WD + O, regardless of the presence or absence of dietary C_20-22_ n-3 PUFA. Overall, this analysis showed that diet-induced changes in hepatic membrane lipid composition are influenced by substrate availability for membrane phosphoglycerolipid synthesis, as well as changes in the expression of enzymes involved in membrane remodeling.

### Oxidized PUFA and lipid peroxidation

**Figure 14:**
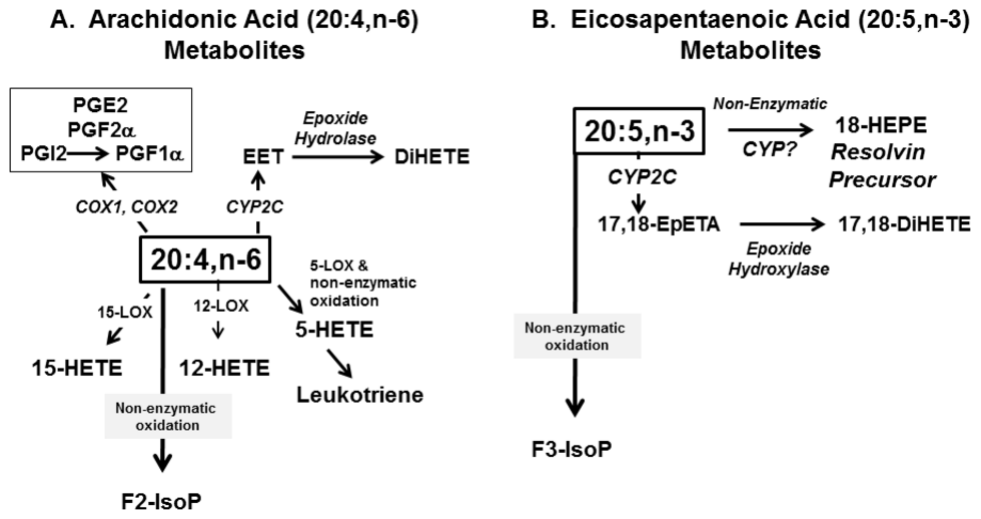
Pathways for the formation of oxidized lipids from arachidonic acid (20:4,n-6) [Panel A] and eicosapentaenoic acid (20:5,n-3)[Panel B]. See text for explanation.

**Figure 15:**
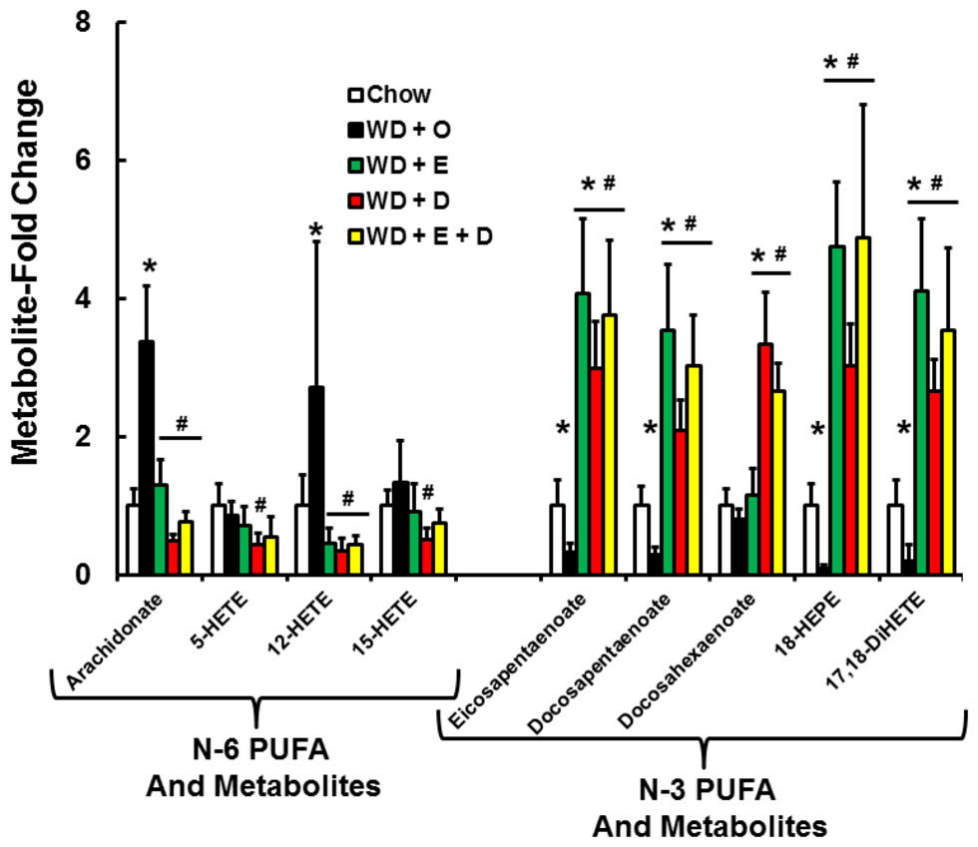
Diet effects on hepatic oxidized lipids. N-6 and n-3 PUFA and oxidized fatty acids were quantified by the metabolomic analysis (Methods). Results are expressed and Metabolite-Fold Change relative to the chow-fed group, mean ± SD, n=8/group; *, *p* ≤ 0.05 versus chow; #, *p* ≤ 0.05 versus WD + O.

PUFA are sensitive to enzymatic and non-enzymatic mono-oxidation (Figure **14**). Enzymatic oxidation involving cyclooxygenases (COX), lipooxygenases (LOX) and CYP2 family members requires excision of the fatty acyl chain from membranes by phospholipases, while non-enzymatic lipid peroxidation likely results from increased hepatic oxidative stress affecting membrane PUFA. The metabolomic analysis identified several oxidized PUFA generated from 18:2,n-6, 20:4,n-6 and 20:5,n-3 (Figure **15**; Tables **S2**-**S4**). The prostaglandin (PG), 6-keto-PGF1α, was only detected in WD + O fed mice. This oxidized fatty acid is generated from 20:4,n-6 by the action of cyclooxygenase and other enzymes; it is a degradation product of PGI2. 5-, 12- and 15-hydroxyeicosatetraenoic acid (HETE) are LOX products derived from 20:4,n-6. The WD + D diet was the most effective diet at suppressing WD-mediated accumulation of oxidized lipids derived from n-6 PUFA. 

Two n-3 PUFA-derived oxidized lipids were detected; 18-HEPE and 17,18-DiHETE. 18-HEPE is derived from non-enzymatic or LOX-dependent mechanisms and is a precursor to resolvins [36]. 17,18-DiHETE is an epoxide hydrolase product of 17,18-epoxyeicosatetraenoic acid (17,18-EpETE); epoxide formation is catalyzed by CYP2 family members. The abundance n-3 PUFA-derived oxidized lipids paralleled changes in hepatic EPA levels (Figure **15**). 

**Figure 16:**
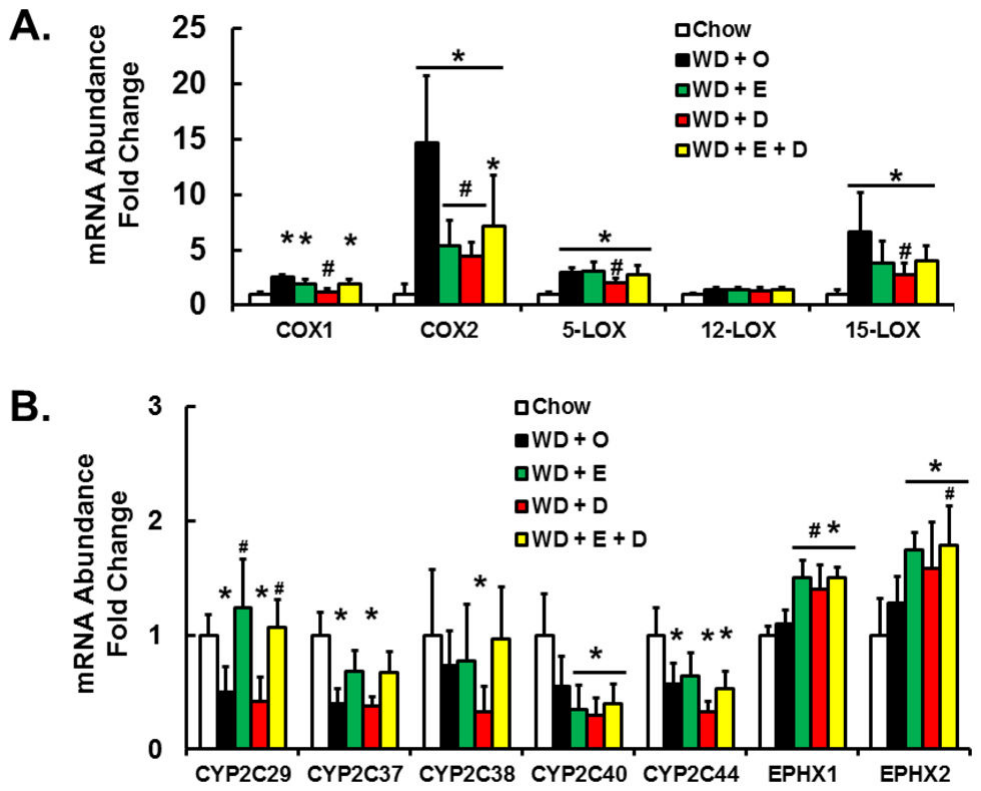
Expression of enzymes involved in fatty acid oxidation. Panel A: Hepatic expression of cyclooxygenases (COX1 & 2), lipoxygenases (5-. 12- & 15-LOX). Panel B: Hepatic expression of cytochrome P450-C2 (CYP2C subtypes) and soluble and microsomal epoxide hydroxylases (EPHX1 and 2). Results are represented as mRNA Abundance-Fold Change, mean ± SD; n=8/group; *, *p* ≤ 0.05 versus chow; #, *p* ≤ 0.05 versus WD + O.

 To determine if changes in the hepatic abundance of these fatty acids are linked to enzyme expression, we quantified hepatic expression of COX1 & 2, LOX-5, -12, -15, several CYP2C family members as well as the soluble and microsomal epoxide hydrolases (EPHX1 & 2) (Figure **16**). Expression of COX-1, COX-2, LOX-5 and LOX-15 were induced by the WD + O and suppressed the WD + D diet (Figure **16 A**). Hepatic expression of several Cyp2C subtypes was suppressed by WD + O. In some cases, e.g., CYP2C29 and CYP2C37, WD + E containing diets reversed the effects of WD + O on these enzymes. Expression of EPHX1 & 2 subtypes was modestly induced by WD + E and WD + D diets (Figure **16B**). Changes in the expression of the CYP2C and EPHX subtypes, however, did not correlate with changes in the 17,18-DiHETE metabolite. 

 In addition to the C_20_-derived oxidized PUFA, several 18:2,n-6-derived oxidized fatty acids were detected, including 9,10-hydroxyoctadecenoic acid (9,10-DiHOME) and 9- and 13-hydroxyoctadecadienoic acids, i.e., isobar: 9-HODE and 13-HODE. While 9,10-DiHOME is formed in the CYP2C/EPHX pathway, 9-HODE and 13-HODE are derived through non-enzymatic mechanisms. When compared to chow-fed mice, the isobar representing 9-HODE and 13-HODE was suppressed in livers of all mice fed the WD, regardless of the presence or absence of C_20-22_ n-3 PUFA. 9,10-DiHome, in contrast, was suppressed >80% in the WD + O group only. For many of the oxidized fatty acids detected in the liver, the expression pattern of the enzymes involved, e.g., CYP2C and EPHX subtypes, does not parallel the hepatic abundance of the oxidized lipid suggesting the formation of these oxidized lipids is driven more by substrate availability than enzyme expression. Table **S4** provides a correlation analysis between precursor and product indicating a strong association between polar lipid fatty acid availability and the hepatic abundance of the oxidized lipids. 

### Oxidative Stress and methylglyoxal metabolism

**Figure 17:**
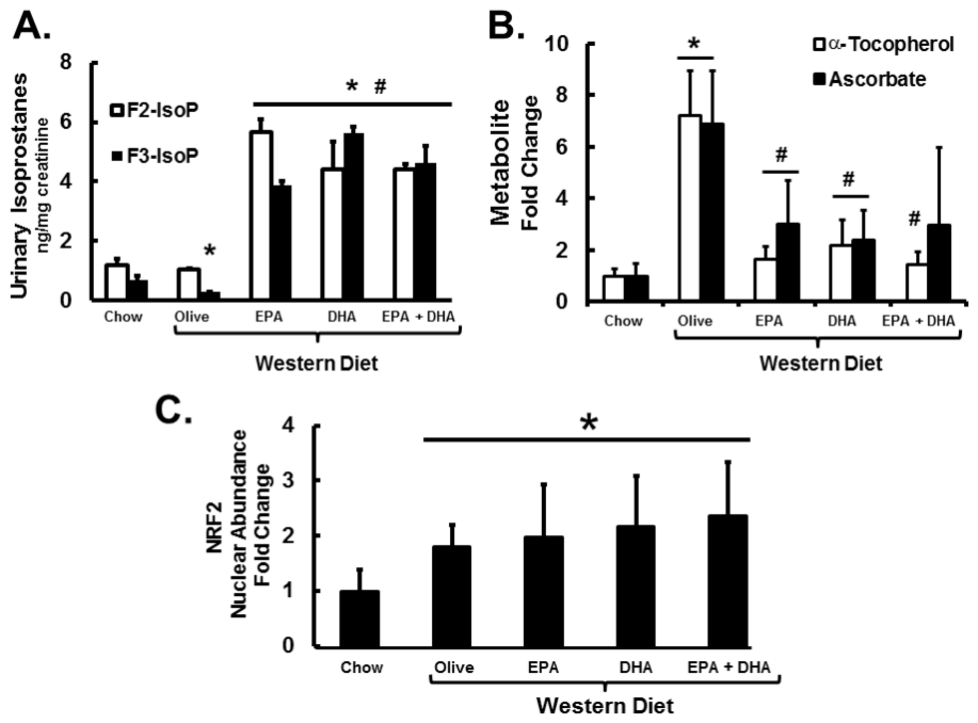
Diet effects on urinary isoprostanes and hepatic α-tocopherol, ascorbate and Nrf2. Panel A: Levels of 24-Hour urinary F2- and F3-IsoPs were quantified as described [17]. F2-IsoPs are derived from arachidonic acid (20:4 n-6) and F3-IsoPs are derived from eicosapentaenoic acid (20:5 n-3) (Fig. 14). Results are represented as Urinary Isoprostanes (ng/mg creatinine) mean + SD, n=3; urine from 2 pools of mice (3 mice/pool) from each diet group were assayed. Panel B: Hepatic α-tocopherol (vitamin E) and ascorbate (vitamin C) were quantified by the metabolomic analysis (Methods) and represented as Metabolite-Fold Change, relative to chow-fed mice; mean ± SD; n=8/group. Panel C: Hepatic nuclear abundance of Nrf2. Hepatic nuclear extracts were assayed for Nrf2 and the loading control protein, TATA-binding protein (TBP) using methods previously described [17]. Nrf2 nuclear abundance was normalized to TBP for each sample. Results are represented as Nrf2 Nuclear Abundance-Fold Change, mean + SD, n=8 group; *, *P* ≤ 0.05 versus chow; #, *P* ≤ 0.05 versus WD + O.

WD diets supplemented with EPA and/or DHA induced the formation of methionine sulfoxide in liver (Figure **7**), a cellular marker of oxidative stress. We previously reported that LDLR^-/-^ mice fed high fat-high cholesterol diets supplemented with menhaden oil had elevated urinary levels of F2- and F3-IsoP and neuroprostane-F4 (NP4) [17]. These iso- and neuroprostanes are derived from 20:4,n-6, 20:5,n-3 and 22:6,n-3, respectively. Herein, we quantified urinary F2- and F3-IsoP. When compared to chow-fed mice, mice fed WD + O had depressed urinary F3-IsoP, but no change in F2-IsoP (Figure **17 A**). Inclusion of EPA or DHA in the WD significantly increased formation and excretion of both F2-IsoP and F3-IsoP. These results are in agreement with our previous study [17] and they indicate that dietary C_20-22_ n-3 PUFA induced isoprostane excretion, reflecting increased whole body lipid peroxidation. 

 The metabolomic analysis showed that LDLR^-/-^ mice fed the WD + O had elevated hepatic α -tocopherol (vitamin E) and ascorbate (vitamin C) [~7-fold, p<0.05] when compared to chow-fed mice (Figure **17B**). This increase was not due to increased dietary vitamin E or C. In fact, vitamin E content was independently quantified in both liver and the diets, and these analyses indicated that the chow diet and all western diets (WD) contained equivalent levels of vitamin E. When vitamin E was normalized to hepatic triglyceride content (hepatic vitamin E/mg triglyceride), hepatic vitamin E in the chow and WD + O groups was not different. In the WD + C_20-22_ n-3 PUFA groups, however, vitamin E was decreased by >60%. Thus, hepatic abundance of EPA and DHA affects hepatic vitamin E and C status. Since decreased hepatic vitamin E and C could promote oxidative stress, we quantified the nuclear abundance of nuclear factor-E2-related factor-2 (Nrf2), a major transcription factor involved in the anti-oxidant response (Figure **17C**). Hepatic nuclear abundance of Nrf2 was increased ≥ 2-fold (p<0.05) in all mice consuming the WD, regardless of the presence or absence of C_20-22_ n-3 PUFA. As such, the WD induced an anti-oxidant response as reflected by increased hepatic nuclear content of Nrf2. Changes in nuclear Nrf2, however, do not parallel changes in hepatic vitamin C or E. 

**Figure 18:**
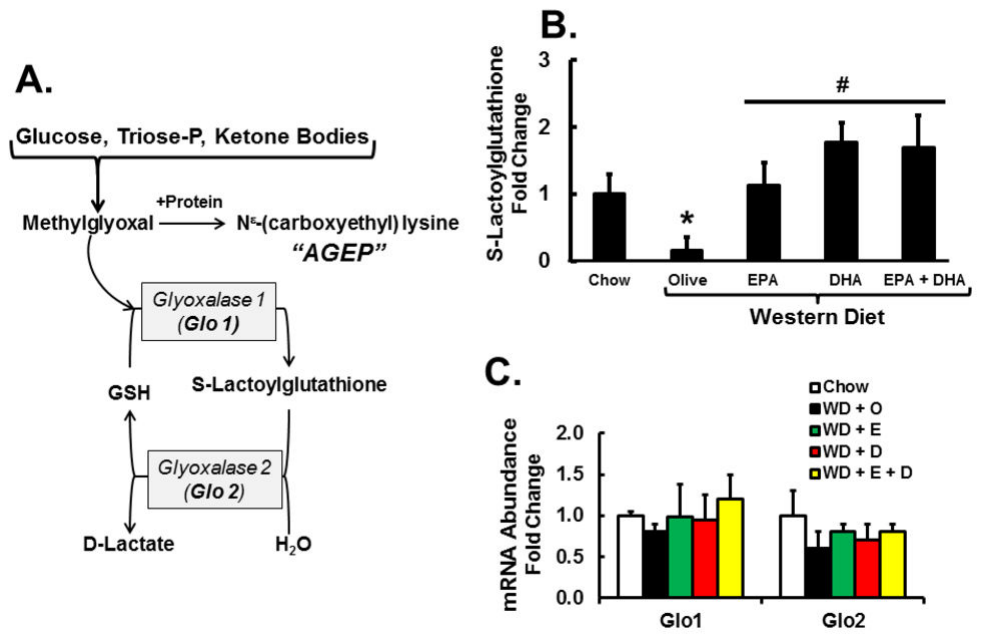
Diet effects on S-lactoylglutathione and metabolites from carbohydrate and lipid oxidation. Panel A: Pathway of methylglyoxal formation and detoxification. Panel B: Hepatic abundance of S-lactoylglutathione was quantified by the metabolomic analysis (Methods). Results are represented as S-Lactoylglutathione-Fold Change, relative to chow-fed mice; mean ± SD, n=8/group. Panel C: Expression of enzymes involved in S-lactoylglutathione formation and degradation, i.e., glyoxalase 1 (Glo 1) and glyoxalase 2 (Glo 2). Results are represented as mRNA Abundance-Fold Change, relative to chow-fed mice; mean + SD, n=8/group.

 While hepatic GSH and GSSH levels were not significantly affected by diet, a glutathione metabolite was significantly affected, i.e., S-lactoylglutathione. S-lactoylglutathione is a detoxification product of methylglyoxal (Figure **18**); and methylglyoxal is involved in the formation of advanced glycation end products (AGEP) and is implicated in fructose induced NASH [23,37]. Increased formation of S-lactoylglutathione is associated with more efficient detoxification of methylglyoxal and decreased AGEP formation. While the hepatic level of S-lactoylglutathione was depressed by 83% (*P* < 0.05) in WD + O fed mice relative to controls, mice fed the WD + C_20-22_ n-3 PUFA diets had ≥ 400% (*P* < 0.05) increase in S-lactoylglutathione (Figure **18B**). 

**Figure 19:**
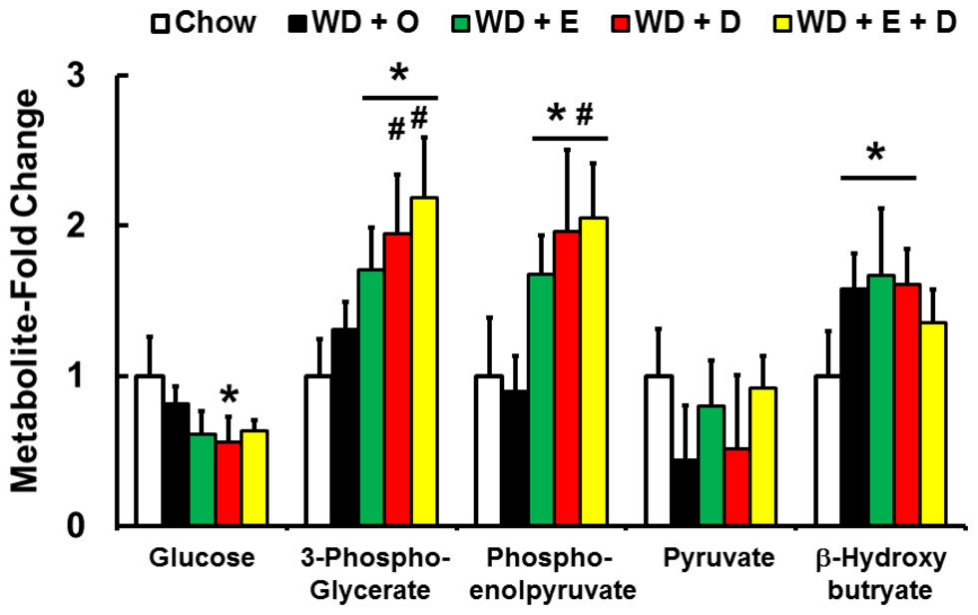
Diet effects on hepatic abundance of glycolytic metabolites that can be converted to methylglyoxal and S-lactoyl-glutathione. Hepatic metabolites were quantified by the metabolomic analysis (Methods) and represented as Metabolite-Fold Change, relative to chow-fed mice; mean ± SD, n=8/group; *, *P* ≤ 0.05 versus chow; #, *P* ≤ 0.05 versus WD + O.

 Factors governing hepatic S-lactoylglutathione are determined by substrate availability and enzyme activity (Figure **18A**). Two enzymes, glyoxalase-1 & -2, are involved in the formation and degradation of S-lactoylglutathione. Expression of neither glyoxalase subtype was affected by diet (Figure **18C**). Of the metabolites affecting methylglyoxal formation [23], hepatic glucose falls and both 3-phosphoglycerate and phosphoenolpyruvate were increased in mice fed the WD + C_20-22_ n-3 PUFA diets (Figure **19**). These outcomes suggest that dietary C_20-22_ n-3 PUFA improves methylglyoxal detoxification by controlling substrate availability. 

## Discussion

 We used a non-targeted global metabolomic approach to gain insight into mechanisms controlling C_20-22_ n-3 PUFA attenuation of WD-induced NASH in LDLR^-/-^ mice. Both WD and dietary C_20-22_ n-3 PUFA induced profound changes in metabolites linked to all major hepatic pathways (Figures **1**-**3**; Table **1**). In this report, we focused on changes in hepatic lipid, amino acid and vitamin metabolism. The accumulation of hepatic sphingomyelin, SFA, MUFA and n-6 PUFA, coupled with depletion of n-3 PUFA, likely plays a major role in NASH-associated pathologies, including hepatosteatosis, inflammation, oxidative stress and fibrosis. The capacity of dietary C_20-22_ n-3 PUFA to lower hepatic sphingomyelin, SFA, MUFA and n-6 PUFA as well as lower hepatic nuclear abundance of NFκB can explain many of the effects of n-3 PUFA on NASH-linked inflammation. 

**Table 1:** Summary of diet effects on *Ldlr* ^*-/-*^ mice.

**Parameter**			**WD + O versus Chow**	**WD + D versus WD + O O**
Plasma Markers:				
Plasma Endotoxin			Increase	No Change
Plasma AST			Increase	Decrease
NASH Markers				
Steatosis			Increase	Partial decrease
Inflammation (MCP1, CD68, TLR4)			Increase	Decrease
Fibrosis (proCol1a1, TIMP1)			Increase	Decrease
Trichrome staining			Increase	Decrease
Oxidative Stress Markers:				
α-Tocopherol			Increase	Decrease
Ascorbate			Increase	Decrease
GSH			No Change	No Change
GSSH			No Change	No Change
S-Lactoylglutathione			Decrease	Increase
Methionine Sulfoxide			No Change	Increase
HMOX1 Expression			Increase	No Change
NOX2 Expression			Increase	Decrease
Urinary Isoprostanes			Decrease, F3-IsoP	Increase F2 & F3-IsoP
Fatty Acid Abundance:				
SFA			Increase	No Change
MUFA			Increase	Decrease
N-6 PUFA			Increase	Decrease
N-3 PUFA			Decrease	Increase
Sphingomyelin			Increase	Decrease
Phospholipids			Enriched in MUFA	Decrease in MUFA
			and N-6 PUFA	and N-6 PUFA
			Depleted of N-3 PUFA	Increased in N-3 PUFA
Oxidized Fatty Acids:				
n-6 PUFA derived			Increase	Decrease
n-3 PUFA derived			Decreased	Increase
Transcription Factors:				
NFκB-p50			Increase	Decrease
NFκB-p65			Increase	No Change
SREBP1			Increase	Decrease
PPARγ2			Increase	No Change
Nrf2			Increase	No Change

A major focus of our analysis was an assessment of metabolic factors that contribute to hepatic inflammation. In the WD-LDLR^-/-^ mouse model of NASH, hepatosteatosis is characterized by a massive accumulation of SFA and MUFA and a significant accumulation of n-6 PUFA, palmitoyl-sphingomyelin and cholesterol. Progression from hepatosteatosis to NASH requires mechanisms that promote inflammation and oxidative stress. Possible sources of inflammatory signals in this model include the high cholesterol diet which promotes high blood cholesterol levels [8]. This likely increases the formation of ox-LDL; and uptake of ox-LDL by Kupffer cells induces secretion of inflammatory cytokines [24]. A second source of inflammatory agents is endotoxin from the gut [25,26]. The increase in plasma endotoxin may be due to increased gut permeability or co-transport with chylomicron [26,29,30]. The WD + O diet induces plasma endotoxin ~15-fold (Figure **4**). Whether this is due to loss of intestinal barrier function or increased endotoxin co-transport with chylomicron requires further study. Regardless of the cause, the cellular target of endotoxin is CD14, the plasma membrane receptor linking TLR4 to NFκB signaling. Once activated, NFκB subunits accumulate in nuclei and induce the transcription of multiple genes involved in inflammation, including MCP1 and TNFα, as well as the induction of multiple cell-surface markers (CD68, Clec4f, Clec10a, F4/80) on Kupffer cells and non-Kupffer leukocytes infiltrating the liver [8]. Inflammation promotes cell death, i.e., necroinflammation, cell debris accumulating from dying cells promotes inflammation by activating TLRs and other damage associated molecular pattern receptors associated with inflammasomes; these events exacerbate the inflammatory response [38]. 

 WD + O induces several hepatic components involved in TLR signaling, including TLR2, TLR4, TLR9, CD14, but not MD2 or MyD88. WD + O also induced IL1β and the inflammasome marker [non-like receptor protein-3, NLRP3 (not shown)]. Dietary C_20-22_ n-3 PUFA attenuated expression of WD-induced TLRs and their components, plus IL1β and NLRP3 [8]. As plasma membrane associated proteins, CD14 and TLR4 function is governed by the composition of lipid rafts; membrane microdomains enriched in sphingomyelin, cholesterol and phospholipids enriched in SFA [39]. The WD + O increased hepatic sphingomyelin 6-fold; this likely increased membrane sphingomyelin and improved TLR4 activation of NFκB signaling in response to inflammatory signals like endotoxin. Increased sphingomyelin correlates with increased sphinganine abundance and increased expression of SPTLC1, 2 and SGMS1. These findings suggest WD increases hepatic sphingomyelin production.

 Dietary EPA and DHA attenuates hepatic inflammation, at least in part, by suppressing *de novo* SFA, MUFA, and sphingomyelin production; this is achieved by suppressing substrate availability (citrate) and the expression of enzymes involved in these pathways (FASN, ACL, SCD1, SPTLC1, SGMS1) (Figures **6**, **8**, **9**). Increased C_20-22_ n-3 PUFA consumption enriches membranes with n-3 PUFA and lowers n-6 PUFA content (Figure **11**). These changes are known to disrupt lipid rafts [40]. Such changes in membrane composition are also associated with suppressed TLR4 and nuclear NFκB content in C_20-22_ n-3 PUFA fed mice [8,17]. While the effects of EPA and DHA on NFκB nuclear abundance and inflammation are well established [33], the impact of EPA and DHA on sphingomyelin and membrane MUFA levels are novel. These studies establish that neither EPA nor DHA affect WD + O-induced plasma endotoxin. Instead, EPA and DHA attenuate hepatic inflammation, at least in part, by inhibiting the cellular response to the inflammatory stimulus, i.e., cytokines and plasma endotoxin. 

The second major focus of our analysis was the effect of diet on hepatic and urinary oxidized lipids and markers of oxidative stress. Our previous studies indicated that high fat-high cholesterol diets induced changes in hepatic markers of oxidative stress [8,17]; including Nrf2, GSTα1, HMOX1 and components of the NOX pathway (NOX1, NOXA1, NOXO1, NOX2, P22phox, P40phox, and P67phox). Oxidative stress has been a target for therapy in patients with NAFLD/NASH; and clinical studies have included both adults and children [41,42].

 In the transition from the chow to the WD + O, hepatic triglyceride, n-6 PUFA, cholesterol, α-tocopherol, ascorbate, and Nrf2 nuclear content increase, while hepatic levels of n-3 PUFA, n-3 PUFA-derived oxidized lipids, and S-lactoylglutathione decrease. GSH and GSSH levels were not significantly affected by the WD + O (Figures **7**, **15**, **17**, **18**; Table **1**). In the transition from the WD + O to the WD + EPA and/or DHA diets, urinary F2-IsoP, F3-IsoP and hepatic methionine sulfoxide, S-lactoylglutathione, and n-3 PUFA-derived oxidized lipids increase, while hepatic α-tocopherol and ascorbate are decreased. Hepatic GSH and GSSH were not significantly affected by the WD (Figure **7**). Increased cellular C_20-22_ n-3 PUFA promoted lipid peroxidation and thus isoprostane formation (Figure **17**) [17]. The decline in α-tocopherol and ascorbate is consistent with increased lipid peroxidation and isoprostane formation; α-tocopherol plays a role in protecting cells from lipoperoxide formation; while ascorbate, which is synthesized in mice, but not humans, plays a role in α-tocopherol recycling [43,44]. More important, however, is that the increased formation of n-3 and n-6 PUFA-derived isoprostanes, methionine sulfoxide and S-lactoylglutathione was associated with decreased gene expression markers of NASH [8]. This raises the question of whether n-3 derived isoprostanes/neuroprostanes and other n-3 PUFA derived oxidized fatty acids are hepatoprotective.

 Our studies also reveal a strong correlation between hepatic content of EPA and DHA and the formation of 18-HEPE and 17,18-DiHETE. 18-HEPE is derived from EPA by a non-enzymatic process or a LOX-dependent mechanism. 18-HEPE is a precursor to resolvins, oxidized fatty acids with anti-inflammatory pro-resolving activity [36]. The epoxide hydroxylase product of 17,18-EpETE is 17,18-DiHETE; and 17,18-EpETE has recently shown to have cardioprotective effects [45]. While the metabolomic analysis did not identify 17,18-EpETE, we have recently detected and quantified 17,18-EpETE and 19,20-epoxy-docosapentaenoic acid (derived from DHA), and the corresponding epoxide hydrolase-derived diols. Using fractionated lipids (Figure **11**; Figure **S1**) and LC/MS methods, we have identified both epoxy- and dihydroxy fatty acids derived from 20:4, n-6, 20:5,n-3 and 22:6,n-3. The epoxy fatty acids are found predominantly in the phospholipid fraction, while the diol fatty acids are found in the non-esterified fatty acid fraction. Cellular levels of these oxidized fatty acids parallel cellular levels of their precursors. Future studies will require determining whether these oxidized fatty acids regulate hepatic cell function. 

### Limitations and Conclusions

There are several limitations to our study. This research used the LDLR^-/-^ mouse and the WD to induce NAFLD/NASH. While the relevance of this model to human NAFLD/NASH has not been established, LDLR^-/-^ mice have provided considerable insight into processes linked to cardiovascular disease. NASH development in these mice is similar, but not identical to that seen in humans. For example, NOX4 induction is a major event in human NASH-associated fibrosis [46]. NOX4, however, is not induced by the WD in LDLR^-/-^ mice. Instead, NOX2 was a major NOX subtype induced by the WD in LDLR^-/-^ mice [8]. This may reflect species differences in how the NOX pathway generates superoxide and hydrogen peroxide, key factors in oxidative stress pathways. While studies investigating DHA supplementation in humans is sparse, we are aware of one study where DHA supplementation had benefit against NAFLD in children [47]. Several clinical trials are underway (6 clinical trials are listed in www.clinicaltrials.gov), but none are comparing the effects of EPA versus DHA on NASH progression. 

 A second limitation is that the metabolomic analysis is designed to detect small molecules. When these small molecules enter other compartments, such as membrane lipids like phosphoglycerolipids, they are not recovered by the extraction methods used for metabolomic analysis. A case in point is the analysis of epoxy and dihydroxy oxidized lipids described above. The identification of 17,18-DiHETE in our study, however, prompted further analysis to identify the precursor to this fatty acid and to search for similar oxidized fatty acids derived from ARA and DHA. 

 A third limitation is that the design of this metabolomic analysis does not allow us to establish cause and effect relationships. The goal of this study was to assess the breadth of effects of the WD, EPA and DHA on hepatic metabolism. In many cases we can relate changes in key pathways, e.g., fatty acid synthesis and inflammation (MCP1), to the control of specific transcription factors (SREBP1 and NFκB). Where there was no information, e.g., sphingomyelin, oxidized lipids or S-lactoylglutathione, we examined expression levels of enzymes involved in these pathways. This approach allowed us to determine whether changes in metabolites correlated with corresponding changes in gene expression. While this approach provides useful information, more studies are required to further define the mechanisms leading to changes in metabolites and expression of enzymes involved in these pathways. 

 Despite these limitations, the outcome of our metabolomic analysis established that the WD and dietary C_20-22_ n-3 PUFA have broad effects on hepatic metabolism affecting all major metabolic pathways. Combined with our gene expression, immunoblot and histological analyses [8], the metabolomic analysis has provided an integrated view of the impact of diet on NASH progression and prevention. Dietary DHA>EPA attenuates NASH progression by regulating multiple processes, including membrane sphingomyelin and phosphoglycerolipid content, nuclear content of key transcription factors (NFκB, SREBP1, NRF2 and others), expression of genes involved in lipid metabolism, inflammation, oxidative stress, fibrosis, improved glucose metabolism, and detoxification of methylglyoxal. In addition, the EPA and DHA containing diets increased the formation of several oxidized lipids that may be hepatoprotective (epoxy- and/or di-hydroxy-fatty acid derivatives of EPA and DHA) (Table **S4**). Mechanisms for EPA and DHA control of membrane lipid content and the nuclear abundance of the transcripts mentioned above have been previously described[33]. The role EPA and DHA play in the control of cellular levels of antioxidants like α-tocopherol and ascorbate, and the role of n-3 PUFA-derived isoprostanes, epoxy- and dihydroxy-fatty acids play in NASH progression is novel. As such, more studies are required to establish how changes in these hepatic metabolites impact NASH progression. Methylglyoxal has been implicated in the progression of NASH [16]. Fructose, a major carbohydrate in the WD, promotes methylglyoxal formation and methylglyoxal is involved in the formation of AGEPs. Finding that addition of DHA to the WD improves methylglyoxal detoxification is equally novel and opens a new window of DHA regulates carbohydrate metabolism. Further definition of these pathways will help explain the sequence of events leading to NASH and its remission in response to dietary C_20-22_ n-3 PUFA. 

## Supporting Information

File S1 **Metabolic data provided by Metabolon, Inc.** Results presented in the excel file are expressed as fold change in comparisons noted at the top of each column. Metabolites that were significantly changed by treatment are: in red if increased and in green if induced. The file also includes the statistical analysis (Welch’s two sample t-test).
(XLSX)

Figure S1 **Thin-layer chromatography of total hepatic and fractionated lipids.** Total lipids were fractionated by solid phase chromatography using an aminopropyl cartridge. Total and fractionated lipids were separated by thin layer chromatography as described in Methods. After separation, lipids were stained with iodine and photographed. Authentic standards (non-esterified fatty acid (NEFA), triglycerides, diacylglycerol phosphatidylcholine (polar lipid), cholesterol and cholesterol esters) were run in adjacent lanes. The chromatogram has three representative hepatic extracts for total lipids, NEFA, polar lipids and neutral lipids.
(TIF)

Figure S2 **Principle component analysis.** Separation of groups by principle component analysis (http://www.metaboanalyst.ca). Known metabolites in the 5 groups [chow (CH); WD + O (WDO); WDE (WD + E); WD + D (WDD); WD + E +D (WDC) were included in the analysis. The explained variances are shown in brackets. The numbers in each bracket/group represents animal identification numbers.
(TIF)

Table S1 **Primer pairs used for qRT-PCR.**
(DOCX)

Table S2 **Volcano plot data comparing Chow versus WD + O fed mice.** Volcano plots were prepared as described in Methods using software at (http://www.metaboanalyst.ca). This table represents the data used to construct Figure 3A.
(DOCX)

Table S3 **Volcano plot data comparing WD + O versus WD + D fed mice.** Volcano plots were prepared as described in Methods using software at (http://www.metaboanalyst.ca). This table represents the data used to construct Figure 3B.
(DOCX)

Table S4 **Correlation of oxidized fatty acids with precursor fatty acids.** Metabolites used to carry out a correlation analysis were quantified by the metabolomic analysis (Methods).
(DOCX)
