# Supplementary figures and images for: A Metabolomic Analysis of Omega-3 Fatty Acid-Mediated Attenuation of Western Diet-Induced Nonalcoholic Steatohepatitis in LDLR -/- Mice
Source: PLoS One. 2013 Dec 17;8(12):e83756. doi: 10.1371/journal.pone.0083756 (PMC3866250; doi:10.1371/journal.pone.0083756)

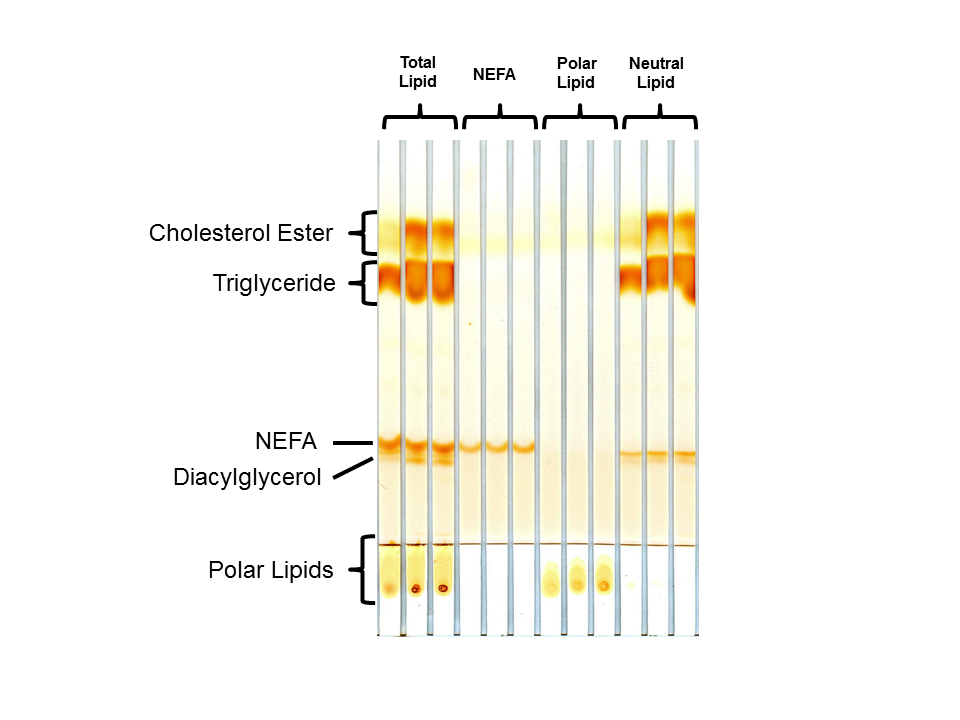

Supplement: Figure S1 — Thin-layer chromatography of total hepatic and fractionated lipids. Total lipids were fractionated by solid phase chromatography using an aminopropyl cartridge. Total and fractionated lipids were separated by thin layer chromatography as described in Methods. After separation, lipids were stained with iodine and photographed. Authentic standards (non-esterified fatty acid (NEFA), triglycerides, diacylglycerol phosphatidylcholine (polar lipid), cholesterol and cholesterol esters) were run in adjacent lanes. The chromatogram has three representative hepatic extracts for total lipids, NEFA, polar lipids and neutral lipids. (TIF) [file pone.0083756.s002.tif]

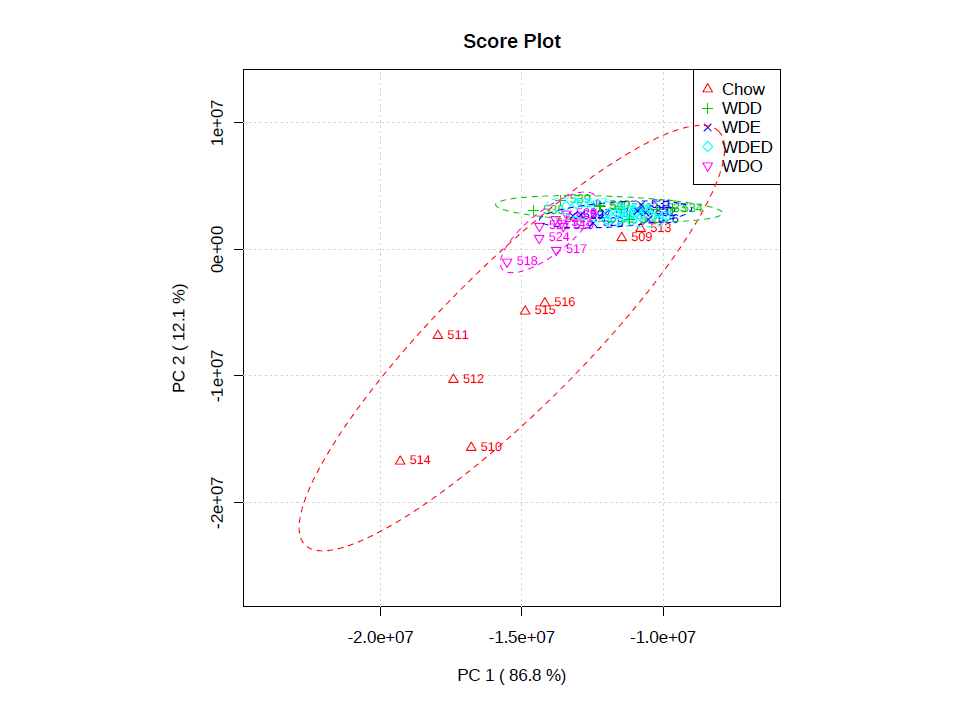

Supplement: Figure S2 — Principle component analysis. Separation of groups by principle component analysis (http://www.metaboanalyst.ca). Known metabolites in the 5 groups [chow (CH); WD + O (WDO); WDE (WD + E); WD + D (WDD); WD + E +D (WDC) were included in the analysis. The explained variances are shown in brackets. The numbers in each bracket/group represents animal identification numbers. (TIF) [file pone.0083756.s003.tif]
