# Supplementary material for: A Metabolomic Analysis of Omega-3 Fatty Acid-Mediated Attenuation of Western Diet-Induced Nonalcoholic Steatohepatitis in LDLR -/- Mice
Source: PLoS One. 2013 Dec 17;8(12):e83756. doi: 10.1371/journal.pone.0083756 (PMC3866250; doi:10.1371/journal.pone.0083756)
Supplement: Table S1 — Primer pairs used for qRT-PCR. (DOCX) [file pone.0083756.s004.docx]

**TABLE S1. Primer pairs used for qRT-PCR.**

| **Transcript** | **Accession #** | **Forward** | **Reverse** |
| --- | --- | --- | --- |
|  |  |  |  |
| Cyclophilin | NM_008907 | CTTCTTGCTGGTCTTGCCATTCCT | GGATGGCAAGCATGTGGTCTTTG |
|  |  |  |  |
| Membrane remodeling (Lands Cycle) |  |  |  |
| PLA2g6 | [NM_001199025.1](http://www.ncbi.nlm.nih.gov/entrez/viewer.fcgi?db=nucleotide&id=312222744) | AAACCCAAGGTGATGCTGAC | CGGTGGCTTCAGGTTAATGT |
| iPLA2 | [NM_026164.2](http://www.ncbi.nlm.nih.gov/entrez/viewer.fcgi?db=nucleotide&id=118130806) | CAGCTGCTCCAGGCTACTTT | CTCTCATACCGTCCTGTGCC |
| LPCAT1 | [NM_145376.5](http://www.ncbi.nlm.nih.gov/entrez/viewer.fcgi?db=nucleotide&id=148747362) | CACGAGCTGCGACTGAGC | ATGAAAGCAGCGAACAGGAG |
| LPCAT2 | [NM_173014.1](http://www.ncbi.nlm.nih.gov/entrez/viewer.fcgi?db=nucleotide&id=27370521) | ACCTGTTTCCGATGTCCTGA | CCAGGCCGATCACATACTCT |
| LPCAT3 | [NM_145130.2](http://www.ncbi.nlm.nih.gov/entrez/viewer.fcgi?db=nucleotide&id=229577258) | AGCCTTAACAAGTTGGCGAC | ATGCCGGTAAAACAGAGCC |
| LPCAT4 | [NM_207206.2](http://www.ncbi.nlm.nih.gov/entrez/viewer.fcgi?db=nucleotide&id=227496911) | GAGTTACACCTCTCCGGCCT | GGCCAGAGGAGAAAGAGGAC |
| Oxidation of Fatty Acids |  |  |  |
| COX1 | [NM_008969.3](http://www.ncbi.nlm.nih.gov/entrez/viewer.fcgi?db=nucleotide&id=144227245) | GCTTGAAGCCTTACACCTCT | TTCTTCAGTGAGGCTGTGTT |
| COX2 | [NM_011198.3](http://www.ncbi.nlm.nih.gov/mapview/maps.cgi?maps=blast_set&db=allcontig_and_rna&na=1&gnl=ref%7CNM_011198.3%7C&gi=118130137&term=118130137%5Bgi%5D&taxid=10090&RID=FSJE9B4B011&QUERY_NUMBER=1&log$=nucltop) | CATATTTGATTGACAGTCCACC | GCTCCTTATTTCCCTTCACAC |
| 5-LOX | [NM_009662.2](http://www.ncbi.nlm.nih.gov/entrez/viewer.fcgi?db=nucleotide&id=116686109) | GCGGGCTGTAGCGAGAAGCA | CTCGCCCTCGCCTGTGATCC |
| 12-LOX | [NM_007440.4](http://www.ncbi.nlm.nih.gov/entrez/viewer.fcgi?db=nucleotide&id=145966724) | CGCCCACCAGCAAGGACGAC | GCGACCCAGGTGCCATGTGA |
| 15-LOX | [NM_009660.3](http://www.ncbi.nlm.nih.gov/entrez/viewer.fcgi?db=nucleotide&id=134948632) | CCCCGGAGACCAGGGATCGG | TCGCCGCCACGTTCAGGATT |
| CYP2C29 | [NM_007815.3](http://www.ncbi.nlm.nih.gov/entrez/viewer.fcgi?db=nucleotide&id=116268124) | TCAGGTCTTTATCCACATCCCTCCC | TGCTGGGTCTTGAGAGAAGAGGGT |
| CYP2C37 | [NM_010001.2](http://www.ncbi.nlm.nih.gov/entrez/viewer.fcgi?db=nucleotide&id=160948607) | ACACGAGGCGTTTCTCACTC | AGGGCTGCTCAGAATCTTTGT |
| CYP2C38 | [NM_010002.3](http://www.ncbi.nlm.nih.gov/entrez/viewer.fcgi?db=nucleotide&id=153791834) | CACTATGGAGACAGAGGTCTA | CCAAATACAGAGTGAAAACG |
| CYP2C40 | [NM_010004.2](http://www.ncbi.nlm.nih.gov/entrez/viewer.fcgi?db=nucleotide&id=254281185) | CAAAGATGCCCAAACGCAA | CAGAGTGAACACAGGGCCATA |
| CYP2C44 | [NM_001001446.3](http://www.ncbi.nlm.nih.gov/entrez/viewer.fcgi?db=nucleotide&id=268834985) | TACGCTTGCTCTCCTGGTTT | GGTCCTCTCCCAAGGAACTC |
| CYP2J5 | [NM_010007.4](http://www.ncbi.nlm.nih.gov/entrez/viewer.fcgi?db=nucleotide&id=142385606) | GAAATGCAAAAGGACCCTGA | GGACTCTCGGTCAGACAAGC |
| EPHX1 | [NM_010145.2](http://www.ncbi.nlm.nih.gov/entrez/viewer.fcgi?db=nucleotide&id=31981782) | CCCCAAGACCCACGGCCTGA | CCTCGCAGTGGCCACCGAAT |
| EPHX2 | [NM_007940.4](http://www.ncbi.nlm.nih.gov/entrez/viewer.fcgi?db=nucleotide&id=406647872) | GCATTGTCACCAACAACTGG | CTGTGTTGTGGACCAGGATG |
| Sphingomyelin Synthesis |  |  |  |
| SPTLC1 | [NM_009269.2](http://www.ncbi.nlm.nih.gov/entrez/viewer.fcgi?db=nucleotide&id=29244576) | CCTCCAACCCACAACATCGT | TGCCAGGCGCTCTTCTAAAT |
| SPTLC2 | [NM_011479.3](http://www.ncbi.nlm.nih.gov/entrez/viewer.fcgi?db=nucleotide&id=142366849) | ATTGGCGCCTTTGGAAGAGA | GAGCACCAGAAAGGCTCAGT |
| SGMS1 | [NM_001168525.1](http://www.ncbi.nlm.nih.gov/entrez/viewer.fcgi?db=nucleotide&id=270483847) | GAAATGAGGCGAACGAATGT | CACCTTCTTGGGTGACCAGT |
| SGMS2 | [NM_028943.5](http://www.ncbi.nlm.nih.gov/entrez/viewer.fcgi?db=nucleotide&id=210147434) | ACACGGCTGTTTTGGTGGTA | CACCAAGCCCGAGACAAGAA |
