# Supplementary material for: A Metabolomic Analysis of Omega-3 Fatty Acid-Mediated Attenuation of Western Diet-Induced Nonalcoholic Steatohepatitis in LDLR -/- Mice
Source: PLoS One. 2013 Dec 17;8(12):e83756. doi: 10.1371/journal.pone.0083756 (PMC3866250; doi:10.1371/journal.pone.0083756)
Supplement: Table S2 — Volcano plot data comparing Chow versus WD + O fed mice. Volcano plots were prepared as described in Methods using software at (http://www.metaboanalyst.ca). This table represents the data used to construct Figure 3A. (DOCX) [file pone.0083756.s005.docx]

**Table S2. Volcano plot data comparing Chow versus WD + O fed mice.**

| **Biochemical Name** | **RNA** | **FC** | **log2(FC)** | **p.value** | **-Log10(p)** |
| --- | --- | --- | --- | --- | --- |
|  | MCP1 | 21.72 | 4.44 | 3.21E-06 | 5.49 |
|  | CD68 | 21.69 | 4.44 | 3.49E-06 | 5.46 |
|  | ProCol1A1 | 17.35 | 4.12 | 1.35E-04 | 3.87 |
| 13-octadecenoate (18:1,n-5) |  | 10.62 | 3.41 | 1.39E-03 | 2.86 |
| 10-nonadecenoate (19:1,n-9) |  | 10.53 | 3.40 | 6.30E-04 | 3.20 |
| cis-vaccenate (18:1,n-7) |  | 10.45 | 3.38 | 1.97E-03 | 2.71 |
| 17-methylstearate |  | 9.33 | 3.22 | 6.99E-04 | 3.16 |
| 1-palmitoylplasmenylethanolamine |  | 8.41 | 3.07 | 7.81E-03 | 2.11 |
| eicosenoate (20:1,n-9 or n-11) |  | 8.16 | 3.03 | 1.21E-03 | 2.92 |
| sphingosine |  | 7.57 | 2.92 | 1.96E-02 | 1.71 |
| alpha-tocopherol |  | 7.22 | 2.85 | 1.33E-05 | 4.88 |
| myristate (14:0) |  | 7.17 | 2.84 | 2.85E-04 | 3.54 |
| ascorbate (Vitamin C) |  | 6.88 | 2.78 | 5.84E-05 | 4.23 |
| 10-heptadecenoate (17:1,n-7) |  | 6.68 | 2.74 | 8.37E-06 | 5.08 |
| oleate (18:1,n-9) |  | 6.59 | 2.72 | 8.37E-03 | 2.08 |
| oleoyltaurine |  | 6.48 | 2.70 | 7.16E-04 | 3.15 |
|  | NOX2 | 6.45 | 2.69 | 7.35E-06 | 5.13 |
| methyl palmitate (15 or 2) |  | 6.25 | 2.64 | 7.40E-05 | 4.13 |
|  | SCD1 | 6.04 | 2.60 | 4.08E-04 | 3.39 |
| sphinganine |  | 5.98 | 2.58 | 5.07E-03 | 2.29 |
| docosadienoate (22:2,n-6) |  | 5.15 | 2.36 | 3.81E-03 | 2.42 |
| dihomo-linoleate (20:2,n-6) |  | 4.83 | 2.27 | 1.31E-03 | 2.88 |
| 5-methyltetrahydrofolate (5MeTHF) |  | 4.55 | 2.19 | 8.01E-06 | 5.10 |
| 1-arachidonoylglycerophosphocholine |  | 4.37 | 2.13 | 3.64E-02 | 1.44 |
| palmitoyl sphingomyelin |  | 4.28 | 2.10 | 8.10E-07 | 6.09 |
| 2-oleoylglycerophosphocholine |  | 4.17 | 2.06 | 2.89E-02 | 1.54 |
|  | TRL4 | 4.05 | 2.02 | 5.44E-04 | 3.26 |
| cysteine-glutathione disulfide |  | 3.86 | 1.95 | 2.89E-04 | 3.54 |
| myristoleate (14:1,n-5) |  | 3.85 | 1.95 | 3.59E-03 | 2.44 |
| palmitoleate (16:1,n-7) |  | 3.76 | 1.91 | 2.73E-05 | 4.56 |
| dimethylglycine |  | 3.64 | 1.86 | 2.94E-04 | 3.53 |
| pentadecanoate (15:0) |  | 3.55 | 1.83 | 4.20E-03 | 2.38 |
| 1-oleoylglycerophosphoinositol |  | 3.47 | 1.80 | 8.59E-03 | 2.07 |
| succinylcarnitine |  | 3.39 | 1.76 | 4.07E-05 | 4.39 |
| guanosine |  | 3.38 | 1.76 | 6.08E-03 | 2.22 |
| arachidonate (20:4,n-6) |  | 3.37 | 1.75 | 3.62E-05 | 4.44 |
| chenodeoxycholate |  | 3.35 | 1.74 | 1.26E-03 | 2.90 |
| phosphoethanolamine |  | 3.33 | 1.74 | 1.34E-03 | 2.87 |
| 1-arachidonoylglycerophosphoinositol |  | 3.33 | 1.74 | 2.03E-02 | 1.69 |
| 1-eicosatrienoylglycerophosphocholine |  | 3.25 | 1.70 | 4.31E-02 | 1.37 |
| 1-oleoylglycerophosphocholine |  | 3.12 | 1.64 | 3.49E-02 | 1.46 |
| adenine |  | 3.09 | 1.63 | 2.85E-04 | 3.55 |
| 2-oleoylglycerophosphoethanolamine |  | 3.06 | 1.61 | 1.04E-03 | 2.98 |
| dihomo-linolenate (20:3,n-3 or n-6) |  | 3.06 | 1.61 | 4.68E-03 | 2.33 |
| 1-pentadecanoylglycerophosphocholine |  | 2.97 | 1.57 | 4.70E-02 | 1.33 |
| 1-oleoylglycerophosphoethanolamine |  | 2.84 | 1.51 | 1.59E-03 | 2.80 |
| 5-methylthioadenosine (MTA) |  | 2.83 | 1.50 | 5.13E-04 | 3.29 |
| gamma-glutamylleucine |  | 2.78 | 1.47 | 6.88E-04 | 3.16 |
| squalene |  | 2.77 | 1.47 | 3.29E-05 | 4.48 |
| 1-myristoylglycerophosphocholine |  | 2.72 | 1.44 | 2.47E-02 | 1.61 |
| aspartylleucine |  | 2.65 | 1.41 | 2.39E-02 | 1.62 |
| gamma-glutamylisoleucine |  | 2.61 | 1.38 | 9.08E-04 | 3.04 |
| taurochenodeoxycholate |  | 2.60 | 1.38 | 1.91E-02 | 1.72 |
| glycerophosphorylcholine (GPC) |  | 2.48 | 1.31 | 3.03E-02 | 1.52 |
| 6-beta-hydroxylithocholate |  | 2.36 | 1.24 | 1.82E-02 | 1.74 |
| beta-muricholate |  | 2.29 | 1.20 | 2.12E-02 | 1.67 |
| N1-methyladenosine |  | 2.21 | 1.15 | 2.91E-05 | 4.54 |
| adrenate (22:4,n-6) |  | 2.20 | 1.14 | 8.51E-05 | 4.07 |
| glycerol |  | 2.19 | 1.13 | 2.02E-02 | 1.69 |
| dihydrocholesterol |  | 2.16 | 1.11 | 1.42E-04 | 3.85 |
| 1-arachidonoylglycerophosphoethanolamine |  | 2.14 | 1.10 | 8.41E-03 | 2.08 |
| 2'-deoxyguanosine |  | 2.11 | 1.08 | 2.32E-03 | 2.63 |
| laurate (12:0) |  | 2.10 | 1.07 | 6.46E-04 | 3.19 |
| S-methylglutathione |  | 2.07 | 1.05 | 1.06E-03 | 2.97 |
| margarate (17:0) |  | 2.07 | 1.05 | 3.00E-03 | 2.52 |
| gamma-glutamylvaline |  | 2.05 | 1.04 | 9.50E-03 | 2.02 |
| 1-linoleoylglycerophosphoinositol |  | 0.50 | -1.00 | 2.71E-02 | 1.57 |
| linolenate [alpha or gamma; (18:3,n-3 or n-6)] |  | 0.50 | -1.01 | 1.44E-02 | 1.84 |
| xanthosine |  | 0.49 | -1.04 | 3.92E-04 | 3.41 |
| glucosamine |  | 0.48 | -1.04 | 9.47E-03 | 2.02 |
| maltose |  | 0.48 | -1.05 | 7.76E-03 | 2.11 |
| propionylcarnitine |  | 0.47 | -1.10 | 1.35E-02 | 1.87 |
| inosine |  | 0.45 | -1.14 | 1.57E-03 | 2.81 |
| pyruvate |  | 0.44 | -1.19 | 5.30E-03 | 2.28 |
| homocysteine |  | 0.39 | -1.35 | 3.62E-02 | 1.44 |
| 13-HODE + 9-HODE |  | 0.39 | -1.37 | 2.32E-04 | 3.63 |
| eicosapentaenoate (EPA; 20:5,n-3) |  | 0.34 | -1.58 | 1.16E-03 | 2.94 |
| 2-linoleoylglycerophosphoethanolamine |  | 0.32 | -1.63 | 4.55E-03 | 2.34 |
| docosapentaenoate (n-3 DPA; 22:5,n-3) |  | 0.29 | -1.77 | 1.10E-04 | 3.96 |
| 7-alpha-hydroxycholesterol |  | 0.27 | -1.86 | 1.90E-04 | 3.72 |
| stearidonate (18:4,n-3) |  | 0.23 | -2.11 | 1.60E-02 | 1.80 |
| 17,18-dihydroxy-5Z,8Z,11Z,14Z-eicosatetraenoate |  | 0.21 | -2.28 | 2.60E-04 | 3.58 |
| 9,10-hydroxyoctadec-12(Z)-enoic acid |  | 0.20 | -2.30 | 8.14E-05 | 4.09 |
| maltotriose |  | 0.19 | -2.40 | 1.71E-02 | 1.77 |
| ergothioneine |  | 0.16 | -2.61 | 1.13E-06 | 5.95 |
| S-lactoylglutathione |  | 0.16 | -2.68 | 1.99E-05 | 4.70 |
| maltotetraose |  | 0.13 | -2.94 | 2.50E-02 | 1.60 |
| 18-HEPE |  | 0.09 | -3.46 | 7.46E-05 | 4.13 |
|  |  |  |  |  |  |
