# Supplementary material for: A Metabolomic Analysis of Omega-3 Fatty Acid-Mediated Attenuation of Western Diet-Induced Nonalcoholic Steatohepatitis in LDLR -/- Mice
Source: PLoS One. 2013 Dec 17;8(12):e83756. doi: 10.1371/journal.pone.0083756 (PMC3866250; doi:10.1371/journal.pone.0083756)
Supplement: Table S3 — Volcano plot data comparing WD + O versus WD + D fed mice. Volcano plots were prepared as described in Methods using software at (http://www.metaboanalyst.ca). This table represents the data used to construct Figure 3B. (DOCX) [file pone.0083756.s006.docx]

**Table S3. Volcano plot data comparing WD + O versus WD + DHA fed mice.**

| **Biochemical Name** | **RNA** | **FC** | **log2(FC)** | **p-value** | **-Log10(p)** |
| --- | --- | --- | --- | --- | --- |
| 18-HEPE |  | 33.31 | 5.06 | 2.19E-06 | 5.66 |
| 17,18-dihydroxy-5Z,8Z,11Z,14Z-eicosatetraenoate |  | 12.95 | 3.69 | 5.11E-08 | 7.29 |
| S-lactoylglutathione |  | 11.37 | 3.51 | 1.14E-08 | 7.94 |
| eicosapentaenoate (EPA; 20:5,n-3) |  | 8.92 | 3.16 | 7.10E-06 | 5.15 |
| docosapentaenoate (n3 DPA; 22:5,n-3) |  | 7.09 | 2.83 | 4.41E-06 | 5.36 |
| 7-alpha-hydroxycholesterol |  | 6.29 | 2.65 | 4.46E-04 | 3.35 |
| docosahexaenoate (DHA; 22:6,n-3) |  | 4.18 | 2.06 | 1.95E-05 | 4.71 |
| 9,10-hydroxyoctadec-12(Z)-enoic acid |  | 4.01 | 2.00 | 3.08E-04 | 3.51 |
| 3-hydroxypyridine |  | 3.14 | 1.65 | 1.02E-02 | 1.99 |
| xylonate |  | 2.83 | 1.50 | 3.19E-03 | 2.50 |
| 7-beta-hydroxycholesterol |  | 2.57 | 1.36 | 6.55E-04 | 3.18 |
| propionylcarnitine |  | 2.25 | 1.17 | 2.35E-02 | 1.63 |
| phosphoenolpyruvate (PEP) |  | 2.19 | 1.13 | 5.89E-04 | 3.23 |
| 5-HETE |  | 0.50 | -1.01 | 2.41E-04 | 3.62 |
| thymidine |  | 0.49 | -1.02 | 1.43E-03 | 2.85 |
| phosphoethanolamine |  | 0.47 | -1.07 | 6.84E-03 | 2.16 |
| taurochenodeoxycholate |  | 0.46 | -1.13 | 3.63E-02 | 1.44 |
| squalene |  | 0.44 | -1.17 | 9.38E-05 | 4.03 |
| dihydrobiopterin |  | 0.44 | -1.18 | 1.57E-03 | 2.80 |
| beta-muricholate |  | 0.44 | -1.20 | 2.19E-02 | 1.66 |
| citrate |  | 0.43 | -1.23 | 5.11E-06 | 5.29 |
|  | TLR4 | 0.42 | -1.26 | 2.34E-03 | 2.63 |
|  | NOX2 | 0.40 | -1.33 | 2.72E-05 | 4.57 |
|  | SCD1 | 0.40 | -1.34 | 3.27E-03 | 2.49 |
| 15-HETE |  | 0.38 | -1.38 | 5.17E-03 | 2.29 |
|  | MCP1 | 0.38 | -1.38 | 1.25E-05 | 4.90 |
|  | CD68 | 0.38 | -1.39 | 1.38E-05 | 4.86 |
|  | ProCol1A1 | 0.36 | -1.48 | 9.78E-04 | 3.01 |
| myristate (14:0) |  | 0.36 | -1.48 | 1.60E-03 | 2.79 |
| linolenate [alpha or gamma; (18:3,n-3 or n-6)] |  | 0.35 | -1.50 | 2.22E-04 | 3.65 |
| 1-eicosatrienoylglycerophosphocholine |  | 0.35 | -1.50 | 4.66E-02 | 1.33 |
| ascorbate (Vitamin C) |  | 0.35 | -1.53 | 2.31E-04 | 3.64 |
| 17-methylstearate |  | 0.35 | -1.53 | 3.96E-03 | 2.40 |
| putrescine |  | 0.33 | -1.59 | 1.45E-02 | 1.84 |
| 1-arachidonoylglycerophosphoinositol |  | 0.31 | -1.69 | 2.16E-02 | 1.67 |
| alpha-tocopherol |  | 0.31 | -1.71 | 1.62E-05 | 4.79 |
| eicosenoate (20:1,n-9 or n-11) |  | 0.29 | -1.79 | 3.81E-03 | 2.42 |
| maltose |  | 0.28 | -1.85 | 4.31E-05 | 4.37 |
| cis-vaccenate (18:1,n-7) |  | 0.24 | -2.04 | 4.98E-03 | 2.30 |
| 10-nonadecenoate (19:1,n-9) |  | 0.24 | -2.08 | 1.70E-03 | 2.77 |
| 2-arachidonoylglycerophosphoethanolamine |  | 0.20 | -2.30 | 1.86E-02 | 1.73 |
| adrenate (22:4,n-6) |  | 0.18 | -2.44 | 6.26E-06 | 5.20 |
| dihomo-linolenate (20:3,n-3 or n-6) |  | 0.18 | -2.47 | 1.64E-03 | 2.79 |
| 1-arachidonoylglycerophosphoethanolamine |  | 0.16 | -2.63 | 6.64E-04 | 3.18 |
| arachidonate (20:4,n-6) |  | 0.15 | -2.78 | 1.75E-05 | 4.76 |
| 2-arachidonoylglycerophosphocholine |  | 0.13 | -2.92 | 1.58E-02 | 1.80 |
| 1-arachidonoylglycerophosphocholine |  | 0.13 | -2.93 | 2.20E-02 | 1.66 |
| 12-HETE |  | 0.13 | -2.96 | 1.54E-02 | 1.81 |
| dihomo-linoleate (20:2,n-6) |  | 0.12 | -3.04 | 7.79E-04 | 3.11 |
| docosadienoate (22:2,n-6) |  | 0.10 | -3.39 | 2.15E-03 | 2.67 |
| maltotriose |  | 0.06 | -4.09 | 5.80E-03 | 2.24 |
