# Supplementary material for: A Metabolomic Analysis of Omega-3 Fatty Acid-Mediated Attenuation of Western Diet-Induced Nonalcoholic Steatohepatitis in LDLR -/- Mice
Source: PLoS One. 2013 Dec 17;8(12):e83756. doi: 10.1371/journal.pone.0083756 (PMC3866250; doi:10.1371/journal.pone.0083756)
Supplement: Table S4 — Correlation of oxidized fatty acids with precursor fatty acids. Metabolites used to carry out a correlation analysis were quantified by the metabolomic analysis (Methods). (DOCX) [file pone.0083756.s007.docx]

|  |  |  |  |  |  |
| --- | --- | --- | --- | --- | --- |

**Table S4. Correlation of oxidized fatty acids with precursor fatty acids^1^.**

**Precursor Fatty Acid Oxidized Fatty Acid Correlation Coefficient^2^**

18:2,n-6 13 + 9-HODE 0.55

18:1,n-6 9.10-DiHOME 0.45

20:4,n-6 6-keto-PGF1α 0.63

20:4,n-6 5-HETE 0.75

20:4,n-6 12-HETE 0.63

20:4,n-6 15-HETE 0.74

20:5,n-3 17,18-DiHETE 0.92

20:5,n-3 18-HEPE 0.92

**^1^**Correlation coefficients were calculated using precursor fatty acid data from the analysis of the hepatic polar lipids and oxidized lipids quantified in the metabolomic analysis (Figures 11 & 15).

**^2^**All p-values were <0.001
